# Heat‐Excitation‐Based Triboelectric Charge Promotion Strategy

**DOI:** 10.1002/advs.202404489

**Published:** 2024-09-14

**Authors:** Xin Xia, Yunlong Zi

**Affiliations:** ^1^ Thrust of Sustainable Energy and Environment The Hong Kong University of Science and Technology (Guangzhou) Nansha Guangzhou Guangdong 511400 China; ^2^ HKUST Shenzhen‐Hong Kong Collaborative Innovation Research Institute Futian Shenzhen Guangdong 518048 China; ^3^ Guangzhou HKUST Fok Ying Tung Research Institute Nansha Guangzhou Guangdong 511457 China

**Keywords:** charge promotion, heat‐excitation effect, thermionic emission, triboelectric charge

## Abstract

The surface charge decay is observed at high temperatures due to thermionic emission, which, however, may not be the only mechanism contributing to the surface charge variation. Here, a triboelectric charge promotion strategy due to the heat‐excitation effect of hot electrons near the fermi level is demonstrated, while the final charge is determined by the balance between thermionic emission and the heat‐excitation effect. It is demonstrated that metals with lower work function exhibit a better heat excitation capability, and polymers with lower fluorine content in molecule chains further boost the charge output, where metal/Kapton pairs demonstrated a charge promotion of over 2 times at the temperature of 383 K with good durability during 90 min measurement. The heat‐excitation effect and charge durability in sliding freestanding‐triboelectric‐layer (SFT) mode triboelectric nanogenerator (TENG) is demonstrated as well, where the energy is promoted by over 3 times and the capacitor charging speed is doubled as well, with an energy promotion from 109.34 to 373 µJ per cycle to successfully trigger a discharger. This work suggests a promising future of the heat‐excitation effect as a new charge promotion strategy for TENG toward different applications in high‐temperature environments.

## Graphical abstract

The surface charge decay happens at high temperatures due to thermionic emission, which, however, may not be the only mechanism contributing to the surface charge variation. Here, a triboelectric charge promotion strategy due to the heat‐excitation effect of hot electrons near the fermi level is demonstrated, which can promote the energy output of a triboelectric nanogenerator over 3 times.

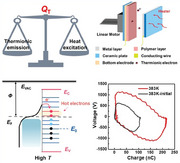

## 1 Introduction

Triboelectrification (or contact electrification, CE) widely exists in daily life when two surfaces contact together with triboelectric charge generated simultaneously.^[^ ^1^ ^]^ Even though triboelectric charge is harmful for the electronics because the extremely high voltage results in electrostatic discharge,^[^ ^2^, ^3^ ^]^ which may damage the electronics and even cause fire, it can be utilized in electrostatic adsorption^[^ ^4^, ^5^, ^6^ ^]^ or dust removal.^[^ ^7^, ^8^ ^]^ Recently, triboelectric nanogenerators (TENGs) suggest a new strategy for employing the electrostatic charge,^[^ ^9^, ^10^ ^]^ which can convert the mechanical stimuli into triboelectricity based on the coupling effects of triboelectrification/CE and electrostatic induction,^[^ ^11^, ^12^, ^13^ ^]^ and has been demonstrated as energy harvesters^[^ ^14^, ^15^, ^16^ ^]^ and self‐powered functional sensors.^[^ ^17^, ^18^, ^19^ ^]^ Thus, it is essential to understand the potential mechanisms contributing to the triboelectric surface charge generation.^[^ ^20^, ^21^ ^]^

The triboelectric surface charge is greatly affected in harsh environments, especially in extreme temperature or humidity conditions.^[^ ^22^, ^23^, ^24^ ^]^ In the uniform‐high‐temperature environment, the surface charge follows an exponential decay due to the thermionic emission,^[^ ^25^, ^26^ ^]^ which served as a method to identify the dominant charge carriers during contact electrification (triboelectric effect) in our previous studies.^[^ ^27^, ^28^, ^29^ ^]^ In the meanwhile, it has been demonstrated that the temperature may also impact contact electrification a lot, while the relationship between the temperature and the charge output is not monotonous.^[^ ^30^, ^31^, ^32^ ^]^ However, the potential mechanism of the temperature effect remains unclear, limiting the applications of TENG in high‐temperature environments. Previous studies demonstrated that the temperature difference can affect both the magnitude and polarity, and hotter materials tend to be positively charged.^[^ ^26^, ^33^ ^]^ Through atomic force microscopy and Kelvin probe force microscopy, the thermionic emission model with temperature differences was revised for nanoscale contact electrification,^[^ ^26^ ^]^ while various factors, especially the material properties, the material size scale, the temperature distribution, etc., still lacks investigation. Considering the electron energy can be excited (by around *k*Δ*T*) through the raised temperature, some scientists studied the optimal Δ*T* of different material pairs, however, the excited surface charge density was much lower than the predicted results, while the mechanism of the heat‐excitation effect still remains unclear.^[^ ^33^ ^]^ Therefore, systematic studies are required to effectively employ the heat‐excitation effect for charge promotion, especially studies on effects from the material properties, such as work functions of metals and fluorine content in polymers which reflects the potential barrier difference during contact electrification and thus contributes to charge generation a lot, targeting to develop the high‐performance TENG at high‐temperature conditions.

Herein, we systematically studied the charge promotion brought by the heat‐excitation effect by different temperatures through contact‐separation (CS) mode TENG as the tool, with the final charge promotion depending on the balance of thermionic emission and heat‐excitation effect. The results demonstrated the widely existence of heat‐excitation effect with metal side heated, and metals with lower work function exhibit a better heat‐excitation capability. Additionally, polymers with lower fluorine content tend to a higher charge promotion, where metal/Kapton pairs demonstrated a charge promotion of over 2 times at the temperature of 383 K with a well durability for 90 min measurement. We also demonstrated the heat‐excitation effect and charge durability in sliding freestanding‐triboelectric‐layer (SFT) mode TENG, where the energy was promoted by over 3 times and the capacitor charging speed was doubled as well. Additionally, we fabricated a SFT mode TENG, with an energy promotion from 109.34 to 373 µJ through heat‐excitation which can trigger the breakdown discharge, suggesting a promising future of heat‐excitation effect as a new charge promotion strategy for TENG toward different applications in high‐temperature environments.

## 2 Results

### 2.1 Experiment Design for Heat‐Excitation Effect

**Figure 1:**
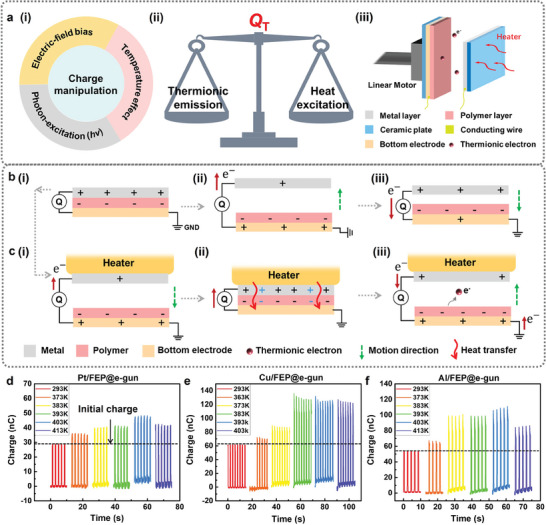
Introduction of the heat‐excitation effect. a) Heat‐excitation‐based manipulation strategy: (i) Charge manipulation strategy in terms of different effects, including the temperature effect; (ii) Temperature effect involving the balance between thermionic emission and heat‐excitation effect; (iii) Schematic diagram of the setup with a single‐side heater on metal side. b) Charge transfer process without heat‐excitation effect. c) Charge transfer process with the heater on the positive side. d–f) Charge variation against temperature of Pt, Cu, and Al contacting with FEP, respectively. The initial charge was controlled as a constant for each pair by the electron‐gun.

Surface charge excitation/regulation of polymers mainly depends on the external excitations, such as the electric‐field bias effect,^[^ ^34^, ^35^ ^]^ photon‐excitation effect^[^ ^36^ ^]^ and temperature effect,^[^ ^31^, ^33^ ^]^ as shown in **Figure** **1**a(i). The temperature effect was generally considered to be the reason of charge decay due to thermionic emission,^[^ ^25^ ^]^ which was employed as a method to investigate the dominant charge transfer mechanism,^[^ ^27^, ^28^, ^37^ ^]^ while the effect of heat for electron‐excitation lacks systematic evaluation. Previous studies demonstrated that a higher temperature may enhance the electron energy levels of metal,^[^ ^26^, ^38^, ^39^ ^]^ and thus it may promote the charge transfer process during contact electrification, which is defined as the heat‐excitation effect in this work. As the result, with the heat input, the surface charge generated by contact electrification may be affected by the balance of the thermionic emission and the heat‐excitation effect, as shown in Figure 1a(ii). When the heat‐excitation effect is dominant, the transferred charge will be enhanced, otherwise charge decay happens due to the thermionic emission. To investigate the relationship between the two effects, a CS mode TENG was employed as the tool and the setup diagram was illustrated in Figure 1a(iii) and Figure S1 (Supporting Information), where the metal side was fixed on a heater. Commonly used metals, aluminum (Al) and copper (Cu), were employed because of their low cost and well performance as electron donors. Additionally, platinum (Pt) was employed to avoid the surface oxidation under high temperature. Polymers with relative high working temperature limit and well electron affinity are preferred, and thus Kapton, polytetrafluoroethylene (PTFE), fluorinated ethylene propylene (FEP), ethylene tetrafluoroethylene (ETFE) are employed.

With the temperature effect on both surfaces considered, the charge transfer without and with one‐side heat‐excitation for metal/polymer pairs are summarized in Figure 1b,c, respectively. Starting from the contact status, when there is no heat source (Figure 1b), through the electrostatic induction by the triboelectric charge, the electrons transfer from the bottom electrode to the top electrode during separation, and then flow back when the two surfaces move to contact again. When the heater is placed at the positive side, the enhancement in potential barrier difference between metal and polymer may boost additional surface charge transfer during contact (Figure 1c(i,ii)). In the meanwhile, there might be thermionic electrons emitted from the negative surface due to the residual heat during separation (Figure 1c(iii)). Thus, the final charge output at high temperature is determined by the balance between the two effects. Based on the setup in Figure 1a(iii), the charge output at varied target temperature *T* of different metals including Pt, Cu and Al, in contact with FEP, were measured and summarized in Figure 1d**–**f, respectively. In each experiment, the initial charge was controlled as a constant for each pair by an electron‐gun (e‐gun) at room temperature. Then, the heater rose to the target temperature at separation status. After that, the charge variation at the target temperature was recorded during continuous CS motions. Details of the experiment setup and operational processes were summarized in Experimental Section. Here, the charge variation against temperature in Figure 1d–f satisfied the charge transfer processes, reflecting the shift of the dominant effect from heat‐excitaion to thermionic emission. The heat‐excitation effect was enhanced with the increase of temperature, leading to a larger charge output. But when the targeted temperature was too high (such as ≈413 K), the charge decay induced by the severe thermionic emission overcame the heat‐excitation effect effect, bringing a lower charge output.

### 2.2 Influence of Metals on the Heat‐Excitation Effect

**Figure 2:**
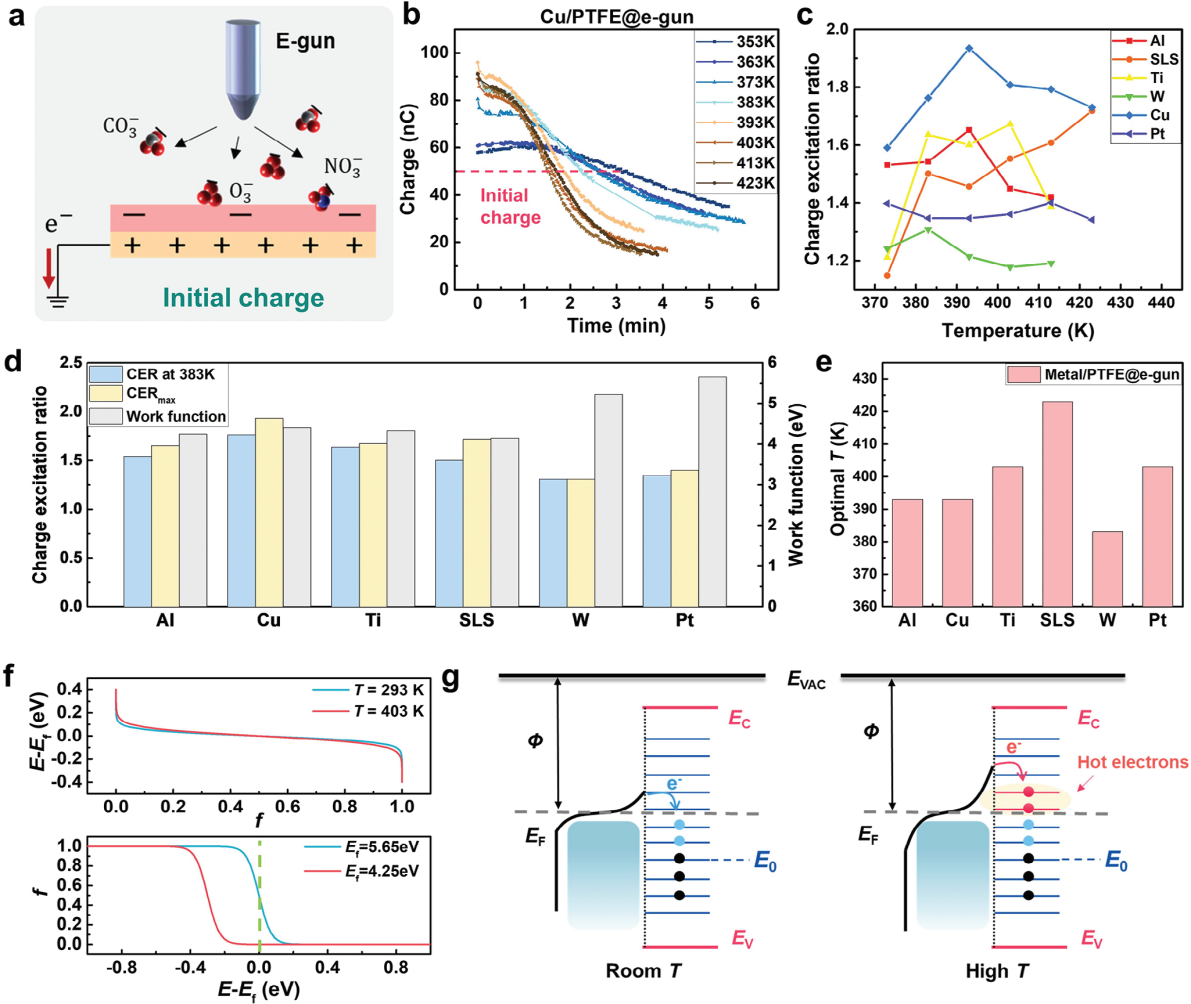
Influence of metals on the heat‐excitation effect. a) Illustration of utilizing e‐gun for the initial surface charge. b) Charge evolution with single side heating of Cu/PTFE. c) Charge excitation ratio summarization of different metals contacting with PTFE. d) Maximized CER (CER_max_) and CER at 383 K of metal/PTFE pairs and the corresponding work function of metals. e) Optimal temperature (*T*_opt_, temperature at CER_max_) of different metals contacting with PTFE. f) Effect of temperature (top) and fermi‐level (bottom) on the fermi‐dirac distribution. g) Contact electrification model at room temperature (left) and with heat‐excitation effect considered (right). Here, the initial surface charge was induced by e‐gun in all mentioned experiments.

As demonstrated in previous studies, the total charge transfer was related to the difference between the work function *Φ* of the two triboelectric surfaces, and the temperature effect on the *Φ* of different materials may be different.^[^ ^10^ ^]^ To better understand the heat‐excitation effect on contact electrification process, different materials and parameters were evaluated. Here, the influence of metals was investigated first, and metals, including Al, Cu, Ti, stainless steel (SLS), W and Pt were employed to contact with PTFE, respectively. To evaluate charge transfer processes with heat and compare the heat‐excitation effect in different materials, the initial surface charge at room temperature is controlled as a constant for each pair by e‐gun (**Figure** **2**a), as depicted in Experimental Section.

Figure 2b summarized the charge evolution at different *T* for the Cu/PTFE with the initial charge ≈50 nC, where an obvious charge enhancement can be observed immediately at the beginning and follows with a severe charge decay, especially at a relatively high *T*. This is because the heat transfer between two surfaces during continuous CS motion promoted thermionic emission. Charge evolution of other metal/PTFE pairs were shown in Figure S2 (Supporting Information), with similar tendency. The decay ratio at 383 K after 3 min was plotted in Figure S3 (Supporting Information), which is defined as the ratio of recorded charge at certain time over the one at the beginning of the measurement with high temperature. To quantify the charge enhancement, a charge excitation ratio (CER) is defined as the ratio of *Q*_T_ over the initial charge. Here, the initial charge is the stable charge induced by different methods at room temperature, and *Q*_T_ was defined as the maximized surface charge measured at the target temperature. Then, *Q*_T_ at different temperature of each material pair was picked out and the corresponding charge ratio was summarized in Figure 2c. With the temperature increase, nearly all charge ratio increased first and then decreased, as the results of the balance between heat‐excitation effect and thermionic emission effect, indicating the shift of the dominant effect from heat excitation to thermionic emission. With continuously increased temperature of SLS/PTFE, charge ratio decayed following the similar trend (Figure S4, Supporting Information). The temperature for peak CER (*T*_opt_) reflected the charge generation capability of metals against temperature, and a higher *T*_opt_ indicated a better charge generation facilitated by the heat‐excitation effect. Surface oxidation on metal surface hinders the charge transfer process and a higher surface hardness like W may cause bad contact intimacy, leading to a worse charge generation under high temperature as well as a lower *T*_opt_. Additionally, the maximized CER (CER_max_) of 1.935 was obtained from Cu/PTFE pair, demonstrating the capability of heat‐excitation effect for charge promotion. However, Pt and W reflected limited charge enhancement capabilities. Considering the temperature effect on the electron energy level of metals, the CER_max_ and the CER at 383 K of each metal was summarized in Figure 2d,e, with the corresponding work function as the reference. The results indicated that metal with a lower work function is prone to a better charge promotion by the heat‐excitation effect, while the one with a higher work function performed worse. Additionally, Figure 2f compared the *T*_opt_ for the CER_max_ of different metal/PTFE pairs, indicating the difference by metals on the heat‐excitation temperature as well. The relationship between work function and corresponding CER can be explained by the fermi‐dirac distribution equation^[^ ^26^ ^]^:

(1) $$f\left(E,T\right)=\frac{1}{e^{\frac{E-E_{F}}{k_{B}T}}+1}$$

Or,

(2) $$f\left\{e^{\frac{E-E_{F}}{k_{B}T}}+1\right\}=1$$

where *f* denotes the probability of an electron in the energy level *E*, *k*_B_ is the Boltzmann constant, *T* is the absolute temperature, *E*_F_ is the fermi level. The equation indicates that when *T*≠0, the probability of electrons with energy lower than fermi level decreases and that with higher energy increases, with total electron numbers remain the same. Thus, partial electrons transit to a higher energy level at a higher temperature to become hot electrons (Figure 2g (top) and Figure 2h (right)). Besides, a higher work function of the metal leads to a smaller *E*_F_ and hence a lower probability of electrons with high energy level (the term of $e^{\frac{E-E_{F}}{k_{B}T}}$ is larger), as reflected by the Equation 2 and Figure 2g (bottom), resulting in fewer hot electrons excited by the heat‐excitation effect, which is consistent with the experimental results. Higher CER_max_ from Cu than Al may result from more severe oxidation of Al, especially at the high temperature condition in air. Generation of Al_2_O_3_ on the Al surface will increase the work function of the electrode side, which reduces the charge generation.^[^ ^40^, ^41^ ^]^ The extremely high surface hardness of W may hinder the contact intimacy and then cause a low CER. Figure 2h shows the physical model at room temperature (left) and at high temperature with heat‐excitation effect considered (right). When the high temperature is employed on the metal side, more hot electrons are generated at the contact interface and transferred from the metal to the polymer surface.

### 2.3 Influence of Polymers on the Heat‐Excitation Effect

**Figure 3:**
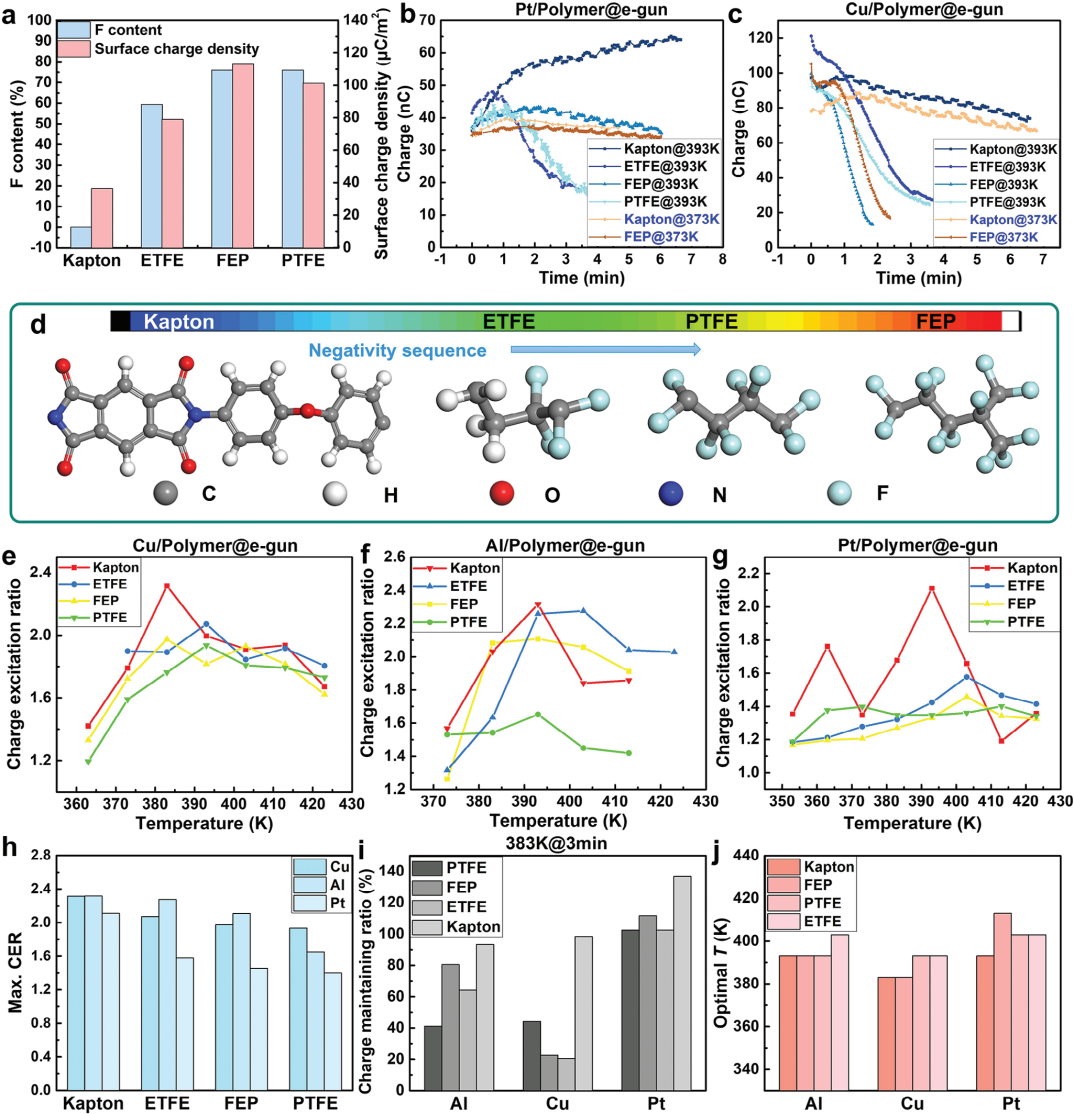
Influence of polymers on the heat‐excitation effect. a) Relationship between fluorine contents of polymers and electron affinity. Charge evolution b) Pt and c) Cu contacting with different polymers at 393 or 373 K. d) Chemical molecule structures of involved polymers and the corresponding electron affinity series. Charge excitation ratio of e) Cu, f) Al, and g) Pt contacting with different polymers at different temperature. h) Maximized CER of different material pairs. i) Charge maintaining ratio of Al, Cu, and Pt contacting with different polymers after 3 min at 383 K. j) Optimal temperature clusters of different material pairs. Here, the initial surface charge was induced by e‐gun in all mentioned experiments.

Besides, influence of polymers was investigated as well. With the same setup illustrated in Figures 1a(iii) and 2a, other polymers including Kapton, ETFE and FEP were utilized as the negative surface. Charge generation capabilities of these polymers were evaluated first, where polymers with a higher fluorine (F) content tends to generate a larger native surface charge density because of the better electron affinity during contact electrification (**Figure** **3**a; Figure S5, Supporting information), indicating a larger potential barrier difference at the contact interface. Chemical molecule structure and the electron affinity sequence of the four polymers were shown in Figure 3d. Figure 3b,c summarized typical charge evolution of Pt and Cu contacting with different polymers, and complete results were plotted in Figure S6 (Supporting Information). The results indicated the dependence of the charge decay on materials. Among all, metal/Kapton pairs always exhibited a well charge durability during heating. Unlike pairs with FEP and PTFE which suffered a severe charge decay with *T* of 393 K, metal/Kapton pairs only reflected a slight charge decay, which might result from the better heat resistance and higher surface hardness of Kapton. Thus, the long‐term durability of the heat‐excited electrons became possible. Additionally, voltage variation was recorded in Figure S7 (Supporting Information), where voltage enhancement was observed as well. CER of Cu, Al, and Pt contacting with the four polymers were summarized in Figure 3e–g, respectively. Here, all material pairs presented the charge promotion under high temperature, where the CERs increased first and then decreased while still are larger than 1, further demonstrating the heat‐excitation effect in various conditions. The excitation voltage ratio of each pair was shown in Figure S8 (Supporting Information), with obvious voltage promotion demonstrated. To better understand the tendency by materials, CER_max_ of each pair was picked out and summarized in Figure 3h, and the CER at 383 K was plotted in Figure S9 (Supporting Information). Here, the results from all polymers follow the conclusion that a lower work function brought a better heat excitation capability. Additionally, the heat excitation capability follows the sequence:

(3) Kapton>ETFE>FEP>PTFE

The sequence indicates that the polymer with lower F‐content may bring a better heat excitation capability, which may benefit from the lower potential barrier height from the polymer with lower F‐content, boosting the enhancement ratio of potential barrier difference. Additionally, the charge output can be doubled by the heat‐excitation effect through proper material selection, e.g. metal/Kapton, Cu/ETFE, etc. The 3 min charge maintaining ratio at 383 K of different tribo‐pairs was shown in Figure 3i. Pt/polymers demonstrated a better charge durability, possibly because of the good oxidation resistance of Pt, where the generation of oxidation on Al and Cu surfaces may hinder the charge generation and cause a charge decay. Attributed to the high surface hardness, Kapton may remove the thin oxide layer in metals during contact separation, bringing better charge durability than that from FEP and PTFE. The charge enhancement along time in Pt/polymer pairs might be attributed to the high work function, where electron transit gradually happens during heating, and then charge transfer is continuously promoted. Figure 3j showed the optimal temperature for the CER_max_ (*T*_op_) of each tribo‐pair, where the temperature for Cu was gathered ≈383 K, a common temperature that can be realized by the industrial heat. Therefore, with material selection in terms of the heat excitation capability, heat stability and target temperature, heat‐excitation effect can be employed as a new charge promotion strategy for triboelectric effect toward different application requirements.

### 2.4 Influence of Initial Surface Charge

**Figure 4:**
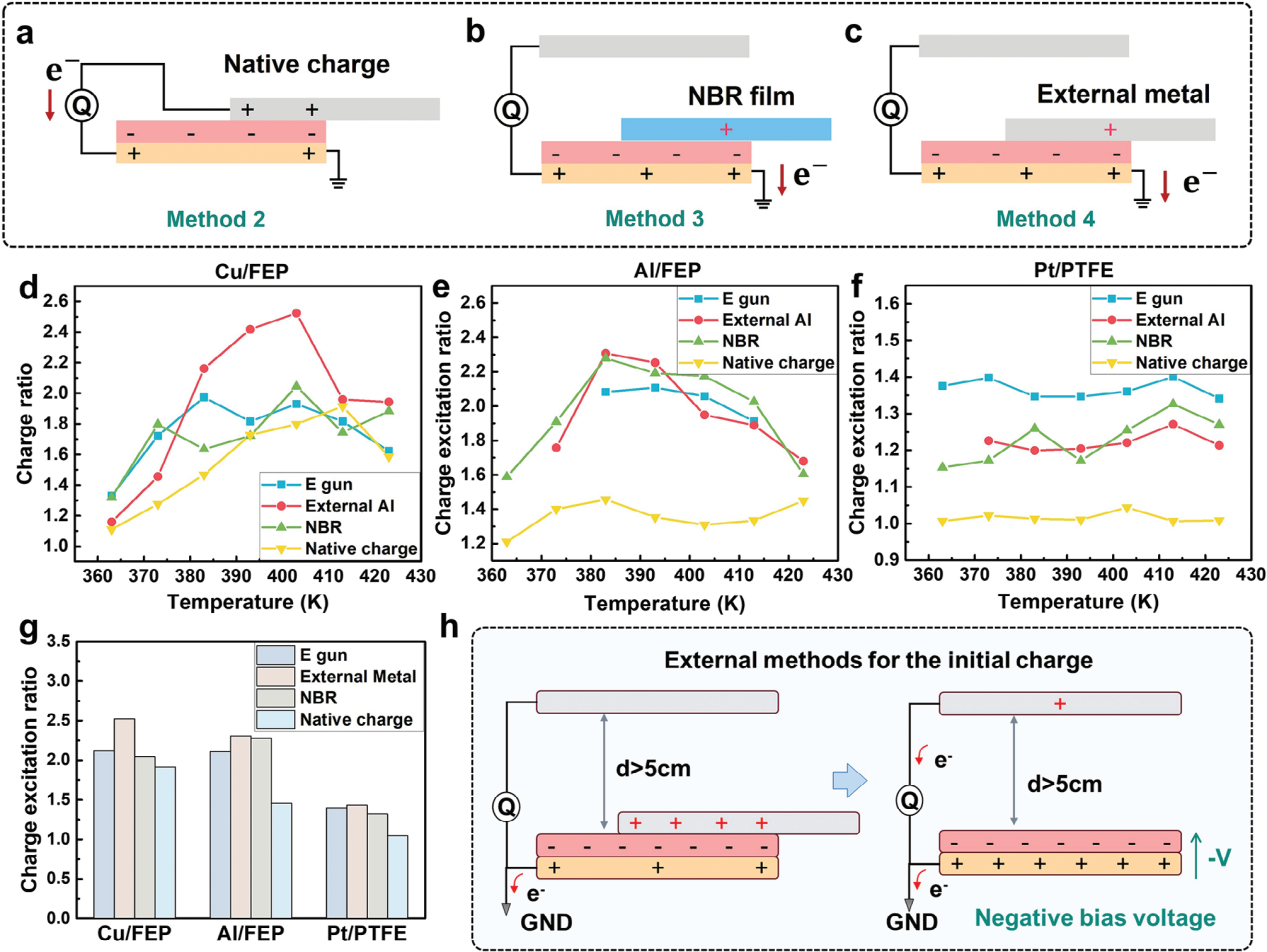
Influence of different initial charge inducing methods. Experiment setup of a) native charge (method 2), b) NBR film sliding on the negative polymer (method 3) and c) identical external metal (method 4) sliding on the negative polymer for inducing the initial surface charge. CER of different charge inducing methods from d) Cu, e) Al and f) Pt contacting with FEP. g) Maximized CER with different charge inducing methods. h) Charge transfer model of external methods for inducing the initial surface charge.

Experiments above employed e‐gun to induce the initial surface charge on the negative surface (as method 1), which may involve the ions during the surface charge transfer process,^[^ ^42^ ^]^ as shown in Figure 2a. To better understand the working mechanism of heat‐excitation effect, we then utilized different types of initial charge introduced by different methods, including native triboelectric charge (method 2) (**Figure** **4**a), charges generated by nitrile butadiene rubber (NBR) layer (Figure 4b) or external metal film sliding on the negative surface (as methods 3 and 4, respectively) (Figure 4c). Cu/FEP, Al/FEP and Pt/PTFE pairs were employed to investigate influence of different initial charge inducing methods, with the CER versus temperature summarized in Figure 4d–f, respectively. Corresponding charge evolution at different temperatures of these tribo‐pairs were shown in Figure S10–S12 (Supporting Information). Here, similar relationship between charge ratio and temperature can be observed, where the charge ratio was first increased and then decreased as the temperature at the metal side increased. Nearly all experiments exhibited an obvious charge promotion by heat regardless of the initial charge inducing methods. Additionally, the results indicated that the tribo‐pair with native charge performed a worse heat excitation capability, as compared to others. Figure 4g picked out the maximized CER of different experimental conditions, which confirmed that charge ratio from native charge was smaller than that from other methods. To exclude the impact of ions induced on the surface through method 1 in Figure 2a, we employed an identical external metal layer to slide on the negative surface with the initial charge in the system induced by method 4. Figure 4g demonstrated that the CER achieved from method 4 was comparable and a little bit higher than that from the method 1 and was much better than that from the method 2. Previous studies demonstrated that the oxides at the interface may elevate the energy level of lowest unoccupied molecular orbital (LOMO) of the polymer, making it difficult for the polymer to attract more electrons.^[^ ^43^ ^]^ Thus, the existence of oxidation air ions including O^−^_3_, O^−^, NO^−^_3_ from method 1 at the interface might attract more electrons and then hinder the charge transfer path between metal and polymer (Figure S13, Supporting Information), resulting in a lower CER_max_ from method 1 than that from method 4. Thus, ions were not the dominant factor that facilitated the heat‐excitation effect. Based on Equation 1, variation of *E*_F_ from different methods only depends on the target temperature, regardless of the charge inducing methods. The final charge transfer is determined by (*E*_F_‐*E*_0_), where *E*_0_ is the energy level of LOMO of the polymer, as shown in Figure 2h, and thus the difference in heat‐excitation capability may be caused by a different *E*_0_ brought by different methods. Charge transfer process of different methods for inducing the initial surface charge were plotted in Figure 4h and Figure S14 (Supporting Information), respectively. It illustrated that during the charge inducing processes, a negative voltage bias was generated at the negative side, which can decrease the LOMO of the polymer^[^ ^30^ ^]^ and finally facilitate the charge transfer processes (Figure S9, Supporting Information). Therefore, external methods (methods 1, 3, and 4) suggested a better heat excitation capability than that by native charge (method 2) in general.

### 2.5 Demonstrations for Applications

**Figure 5:**
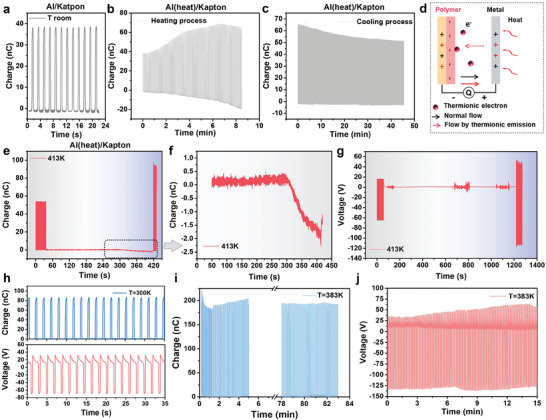
Demonstration of the mechanism and durability. a) Initial charge output of Al/Kapton. Charge variation of Al(heated)/Kapton pair during b) the heating process from room temperature and c) cooling process from the high temperature. d) Circuit diagram for method demonstration. e) Static charge variation of Al(heated)/Kapton pair with zoomed‐in figure in (f). g) Static voltage variation with of Al/Kapton pair. Static charge measurement was realized under the air gap of 15 mm. h) Initial charge (top) and voltage (bottom) of the 5 cm × 5 cm CS mode TENG (Al/Kapton) at room temperature. Durability test of i) charge and j) voltage at 383 K.

To better understand the mechanism of the heat‐excitation effect, the charge evolution of heating and cooling processes was investigated. Considering the stable mechanical and electrical properties as well as the high CER at high temperature, Al/Kapton was utilized. Starting from the initial charge at room temperature (**Figure** **5**a), the charge continuously increased during the heating process until reaching the target temperature (Figure 5b), but when the heater was turned off, the charge decayed during the cooling process as shown in Figure 5c. These results indicated that the heat‐excitation effect can only be maintained by the temperature difference between the triboelectric surfaces, while the effect disappears with the charge decay during the cooling process. Similar heating and cooling behaviors of Cu/Kapton and Al/FEP were demonstrated in Figure S15 (Supporting Information), where the charge increased during heating and then decreased during cooling. However, because of the severe charge decay by thermionic emission in FEP, FEP exhibited a charge enhancement at the beginning and then suffered an even worse charge decay during the continuous heating. To verify the heat‐excitation effect other than the thermionic emission as the reason contributing to the charge promotion, the electrical signals of TENG were measured when the device was statically held at separation status, while the heat‐excitation effect is eliminated, namely the static measurement method. The schematic circuits with hypothesis were illustrated in Figure 5d. The initial charge under continuous CS motion was recorded first. Then, charge variation with the heated metal side was recorded with the TENG held at the separation gap of 15 mm. If the significant charge enhancement was due to the thermionic emission, obvious electron flow or voltage variation for realizing a new electrostatic equilibrium should be recorded at separation status and the thermionic electrons can be partially gathered at the polymer surface, similar to the thermionic energy converter,^[^ ^38^, ^44^, ^45^ ^]^ so the electrons should be transferred from the positive side to the negative side, the same as normal electron flow during separation, as shown in left diagram in Figure 5d. Static measurement results of Al/Kapton pair under different heater locations were illustrated in Figure 5e–g. Here, charge and voltage with nearly no variation were recorded during a long‐time static measurement (6–15 min). When the CS motion of TENG was triggered again after long‐term static measurement, remarkable charge enhancement was recorded. As discussed in Note S1 (Supporting Information), for the Al/Kapton pair at *T* = 413 K, the charge output was enhanced from 55 to 95 nC due to previous experimental results as shown in Figure 5e. If the thermionic emission was the dominant reason for the charge enhancement by the temperature effect, the thermionic emission electrons gathered at the Kapton surface should be at least 40 nC (as the difference between 55 and 95 nC). However, the measured charge variation was only ≈2 nC, demonstrating that the charge enhancement should not only come from the thermionic emission.

The durability of the heat‐excitation effect was evaluated as well. By considering the capability of heat‐excitation and excellent heat stability, Kapton was utilized. A 5 cm × 5 cm Al/Kapton TENG with hard substrate was investigated. When the temperature of the heater increases from 300 to 383 K, the electrical performance was enhanced from 88 nC and 100 V (Figure 5h) to 200 nC and 175 V (Figure 5i,j), where the charge was stable in a 90 min test, as shown in Figure 5i, demonstrating the long‐term capability by the heat‐excitation effect. The severe charge decay in the first several minutes may be attributed to the heat fluctuation before realizing the balance. To demonstrate the heat‐excitation effect in initial high output device output enhancement, a 7 cm × 7 cm Al/Kapton device with silicone substrate was employed with *T* of 373 K, with results summarized in Figure S16 (Supporting Information), where the surface charge was stably enhanced from 380 nC (77.55 µC m^−2^) to 600 nC (122.45 µC m^−2^) by ≈1.57 times during a long‐time measurement larger than 30 min, which is much higher than the previous result of ≈58 µC m^−2^ even under a lower temperature difference.

**Figure 6:**
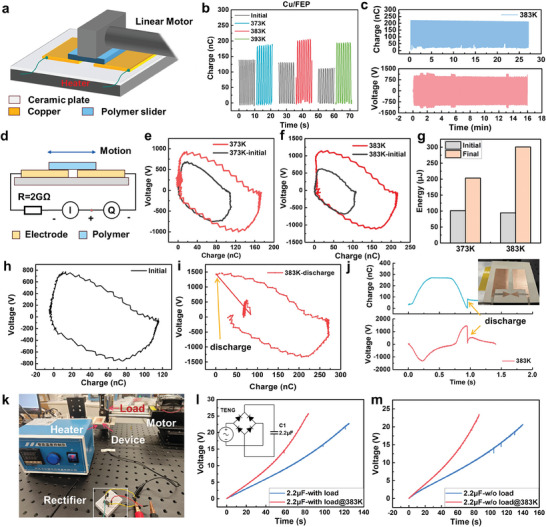
Heat‐excitation effect in SFT mode TENG. a) Schematic diagram of the experiment for SFT mode. b) Charge variation against temperature of Cu/FEP. c) Charge (top) and voltage (bottom) durability test of Cu/FEP pair at 383 K. d) Circuit diagram for CMEO measurement. e,f) CMEO at 373 and 383 K. g) Energy comparison. CMEO of the TENG with discharger at h) initial temperature and i) 383 K with air breakdown. j) Charge and voltage signal with air breakdown at 383 K. Inset was the photograph of the device. k) Photograph of charging experiments. Charging curve of 2.2 µF‐capacitor at 383 K l) with load and m) without load. Here the initial charge was the native charge from the relative sliding of tribo‐pair.

To ensure the capability of heat‐excitation effect in various applications, we also investigated its performance in SFT mode TENG. The experimental setup was illustrated in **Figure** **6**a, where the metal side with a ceramic plate was placed on a heater, and a polymer slider with PU foam substrate was controlled by the linear motor. Details are depicted in Experimental Section. Based on the material properties in CS mode experiments as well as the triboelectrification ability, Cu/FEP were evaluated, and only native charge by method 2 was involved in experiments. Figure 6b showed the charge variation of Cu/FEP at the temperature range of 373–393 K, respectively. Without any cooler design on the negative side for weakening thermionic emission, Cu/FEP suggested a well charge promotion by the heat‐excitation effect and was employed in further demonstrations. The charge limits may be attributed to the severe thermionic emission at the interface. Charge and voltage durability of Cu/FEP at 383 K were recorded in Figure 6c. Charge durability at 373 K was shown in Figure S17 (Supporting Information). Unlike the severe charge decay in Cu/FEP‐based CS mode TENG, Cu/FEP‐based SFT mode TENG exhibited a stable charge output. Here, the initial charge output was only 140 nC (Figure S17, Supporting Information), resulting in a charge promotion ratio of ≈1.43, and the voltage was enhanced from 1240 to 1920 V with well stability, further confirming the charge promotion capability by the heat‐excitation effect. The difference between CS mode and SFT mode devices might contribute to severe surface wear by sliding motions, and thus the oxide layer on Cu may be removed. As discussed in Figure 1a(ii), the final charge output depends on the balance of the thermionic emission effect and heat‐excitation effect. SFT mode TENG was nearly not affected by the thermionic emission for the charge decay, which reflected a well durability. Energy promotion was evaluated as well. Assisted by the measurement circuit in Figure 6d, cycle of effective maximized energy output (CMEO) at different temperature was obtained and compared in Figure 6e,f. The results demonstrated that CMEO was obviously multiplied by the heat‐excitation effect, with the area of CMEO calculated in Figure 6g, where 3‐time energy promotion was realized at *T* of 383 K. Electrical signals during CMEO measurement were shown in Figure S18 (Supporting Information), with well durability. Additionally, we developed a SFT mode TENG with a discharger as the switch (Figure 6j inset), where the discharger only worked when the heat was applied with an energy promotion from 109.34 to 373 µJ (Figure 6h,i), demonstrating the promising applications of heat‐excitation effect by triggering air breakdown. Here, the sudden change in CMEO (Figure 6i) as well as the electrical curve (Figure 6j) reflected the happen of air breakdown. Details of the electrical signal of the SFT mode TENG with discharger was shown in Figure S19 (Supporting Information). Finally, we also demonstrated the charging performance by heat‐excitation effect, with system photograph shown in Figure 6k, with the initial charge and *Q*_T_ shown in Figure S20 (Supporting Information). As reflected in Figure 6l,m, charging speed to a 2.2 µF capacitor was enhanced a lot when the heater was employed, ensuring the capability of heat‐excitation effect toward power promotion applications.

## 3 Discussion

In summary, the heat‐excitation effect was systematically investigated, where the charge promotion was realized when a high‐temperature source was employed on the metal side of TENG. The results demonstrated that the increased electrons’ energy at the contact interface by high temperature brings hot electrons in the interface and then facilitate the charge transfer processes, and the metal with lower work function exhibits a better charge promotion by the heat‐excitation effect. Based on this, a physical model was established. As an important role in heat‐excitation effect, polymers with lower fluorine content tend to a higher charge promotion, where metal/Kapton pairs demonstrated a charge promotion of over 2 times at the *T* of 383–393 K. External initial charge inducing methods, including e‐gun, external metal or NBR layer sliding on the negative surface brings a better charge promotion than native charge. The durability of the heat‐excitation effect was successfully demonstrated, where Cu/Kapton‐based CS mode TENG reflected a stable charge output after 90 min test at the *T* of 383 K. To further confirm the potential applications of the heat‐excitation effect, charge promotion and charge durability was demonstrated in SFT mode TENG, where 3‐time energy promotion was obtained during the CMEO measurement, and capacitor charging speed was doubled as well. Additionally, we developed a TENG with a discharger as the switch, where the discharger can only work with the assistance of heat‐excitation effect, with an energy promotion from 109.34 to 373 µJ, demonstrating the promising applications of the heat‐excitation effect on triggering the air breakdown effect. This work demonstrated the widely existence of the heat‐excitation effect as well as the considerable power promotion capability with well durability, suggesting a promising future of the heat‐excitation effect as a new charge manipulation strategy for the triboelectric effect toward different applications of energy harvesting, self‐powered sensing, etc, especially in harsh temperature environments.

## 4 Experimental Section

### Material Selections

Metal materials, including Al, Cu, Pt, W, Ti and SLS304 were commercial products with thickness of 0.02 mm and purity of 99.99%. Polymers with relative high working temperature limit and well electron affinity were preferred, thus Kapton, PTFE, FEP, ETFE were employed. Here, all polymer films were commercial products with thickness of 50 µm. Al, Cu, and Pt were used to test different polymers. The reason for using Al and Cu was its low cost and well performance as electron donors. Considering the oxidation of Al and Cu at high temperature, Pt was employed to avoid the surface oxidation and serve as a good electron donor.

### Setup of the Experimental Platform

For the CS mode TENG, the metal side was placed on an electrical heater fabricated by cast Al, where a ceramic plate with thickness of 1 mm was placed between the electrode and the heater to ensure heat conduction and electrical insulation. The size of the heater was 15 cm  ×  20 cm  ×  2 cm, with power of 400 W. The temperature of the heater was controlled by a controller through the thermocouple (k‐type) imbedded in the cast Al heater. The negative side of both CS and SFT mode TENG was fixed on the linear motor through 3 m foam tape. The short‐circuit charge transfer (*Q*_SC_) was measured by *Keithley 6514* with sampling rate of 500. Photograph of the experimental platform of CS mode and SFT mode TENG is shown in Figure S1 (Supporting Information), and Figure 6i respectively.

### Operational Process of the Experiment

Three different methods as discussed in Figure 4 were utilized to induce the initial charge on the negative triboelectric surface, including electron‐gun (method 1), NBR‐film (method 3), external metal (method 4) and native charge (method 2). Here, NBR was utilized due to its triboelectric positive polarity and the extraordinarily excellent wear‐resistance as demonstrated in previous study.^[^ ^28^ ^]^ Before inducing charges on the negative surface, the original surface charge was removed through 99.97% ethyl alcohol. Operational process of each method was depicted as follows. For the electron‐gun (method 1), initial charges were directly injected on the surface and the initial charge was recorded until the output was stable. For NBR or external metal layer method (method 3 and 4), the NBR film or the external metal layer slid on the negative surface until the initial charge was realized. The native charge was generated through relative sliding between the two triboelectric surfaces. To investigate the heat‐excitation effect, the initial *Q*_SC_ at room temperature of different tribo‐pairs was controlled at a constant value by the required charge inducing method. After realizing the controlled value of *Q*_SC_, the TENG was hold on at separation condition with air gap ≈50 mm and then the heater started to heat up until the target temperature. Then, the *Q*_SC_ variation of TENG was recorded under continuous CS operations by *Keithley 6514*. *Q*_T_ was defined as the maximized *Q*_SC_ recorded at the target temperature. The operational temperature range was limited by few charges remained on the surface as well as the working temperature of polymers at high temperature, ≈453–473 K for all tribo‐pairs.

### Fabrication of TENG Employed in Different Experiments

The device employed in evaluation for the heat‐excitation effect in Figure 4 was 3 cm × 3 cm CS mode TENG with hard substrate. Metal layer was directly pasted on ceramic substrate (Al_2_O_3_) by Kapton tape. Polymer layers, including Kapton, FEP and PTFE with thickness of 50 µm was also pasted on Al_2_O_3_ plate by Kapton tape. Electrode on the back side of the polymer was silver with thickness of 200 nm deposited by electron‐beam evaporation. Al/Kapton TENG with hard substrate (Al_2_O_3_) applied in Figure 5h–j was fabricated as the same, with effective contact area of 5 cm  ×  5 cm. Fabrication of device with soft substrate (Figure S16, Supporting Information) was a little bit different. Copper tape was utilized as the positive material, which was pasted on a Silicone layer with thickness of 1 mm. Here, the Silicone layer was pasted on the substrate by Kapton tape. The other side (Kapton layer) was directly pasted on the substrate, where the electrode on the back side of the Kapton layer was silver with thickness of 200 nm deposited by electron‐beam evaporation as well. The effective contact area of soft substrate device was 7 cm  ×  7 cm. For the SFT mode TENG, the copper tape was pasted on the 1 mm ceramic plate, with electrode size of 3 cm × 5 cm and an air gap of 6 mm. The substrate of the slider was a 3 cm × 5 cm ceramic plate. Then, a 3 m PU foam with thickness of 2 mm was pasted on the substrate. Finally, the FEP tap with thickness of 80 µm was pasted on the PU foam. The discharger was cut from the copper tape, with an air gap ≈0.8 mm.

## Conflict of Interest

The authors declare no conflict of interest.

## Author Contributions

X.X. conceived the idea. X.X. prepared the samples, fabricated the devices in all experiments. X.X. designed the experiments conducted the electrical performance evaluation. X.X. developed the mechanisms and repeated the main results and Y.Z. participated the data analysis. X.X drafted the paper, Y.Z. revised the paper. All authors participated in the interpretation of the data and production of the final paper.

## Supporting information

Supporting Information

## Data Availability

The data that support the findings of this study are available from the corresponding author upon reasonable request.
