# Supplementary material for: Heat‐Excitation‐Based Triboelectric Charge Promotion Strategy
Source: Adv Sci (Weinh). 2024 Sep 14;11(41):2404489. doi: 10.1002/advs.202404489 (PMC11538680; doi:10.1002/advs.202404489)
Supplement: Supplementary file 1 — Supporting Information [file ADVS-11-2404489-s001.docx]

Supporting Information

Heat-excitation-based triboelectric charge promotion strategy

Xia Xin,^*^ and Yunlong Zi

X. Xia, Y. Zi

Thrust of Sustainable Energy and Environment, The Hong Kong University of Science and Technology (Guangzhou), Nansha, Guangzhou, Guangdong, 511400, China

E-mail: Xin Xia (xinxia@hkust-gz.edu.cn)

Y. Zi

HKUST Shenzhen-Hong Kong Collaborative Innovation Research Institute, Futian, Shenzhen, Guangdong, 518048, China

Y. Zi

Guangzhou HKUST Fok Ying Tung Research Institute, Nansha, Guangzhou, Guangdong 511457, China


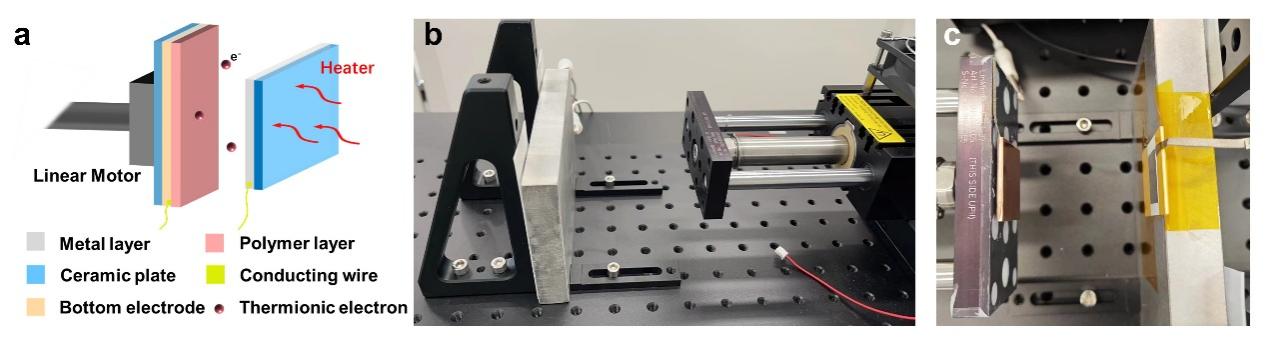


**Figure S1.** Experimental setup a single-side heater on metal side. **a.** Schematic diagram of the setup with a single-side heater on metal side. Photographs of **b.** the experimental setup and **c.** device.


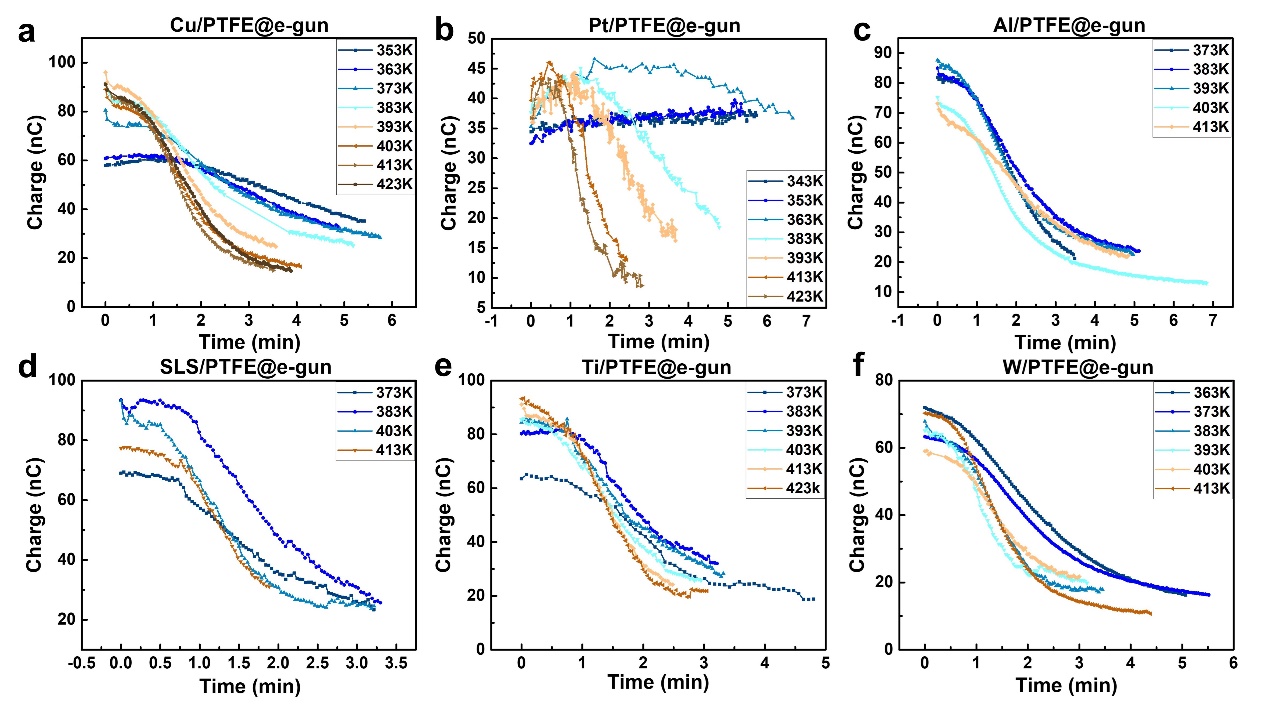


**Figure S2.** Charge evolution of different metal/PTFE pairs. **a.** Cu/PTFE. **b.** Pt/PTFE. **c.** Al/PTFE. **d.** SLS/PTFE. **e.** Ti/PTFE. **f.** W/PTFE. The initial charge was induced by e-gun.


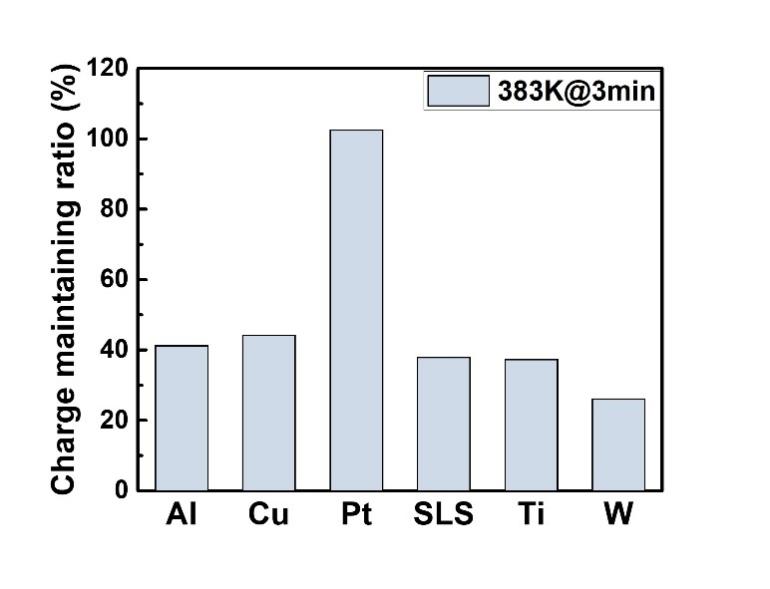


**Figure S3.** 3-minutes charge maintaining ratio at 383K of different metals contacting with PTFE.


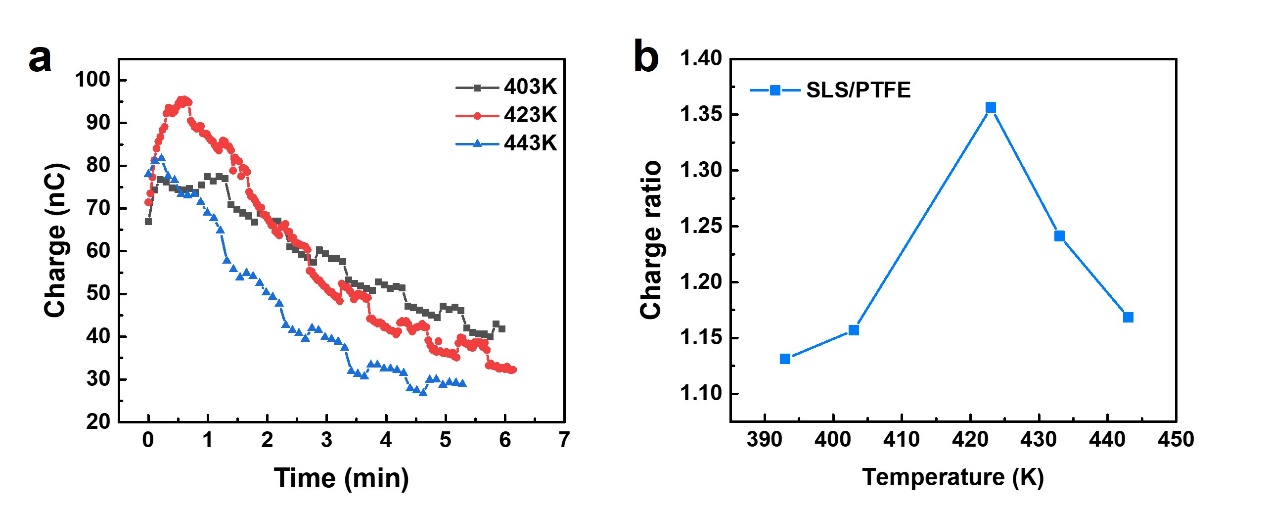


**Figure S4**. **a**. Charge variation and **b**. charge excitation ratio against temperature of SLS/PTFE pair. The initial charge was induced by e-gun.


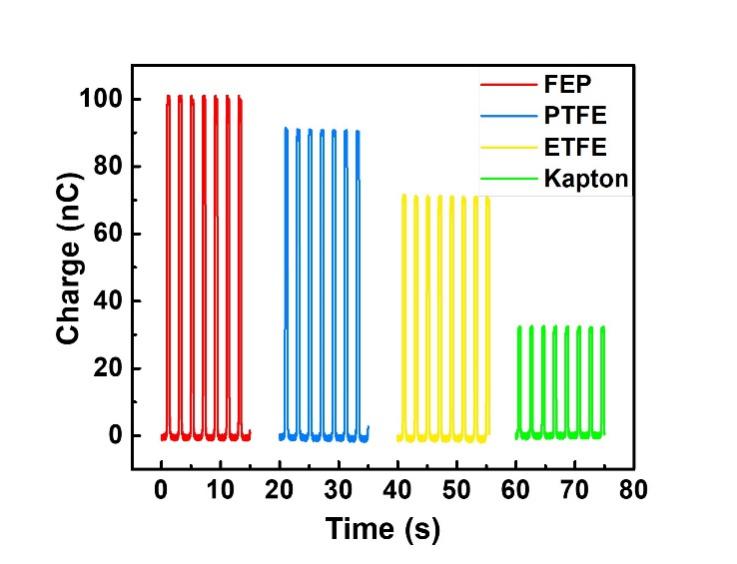


**Figure S5.** Native charge of Cu/polymer pairs. A higher charge output means a better electron affinity.


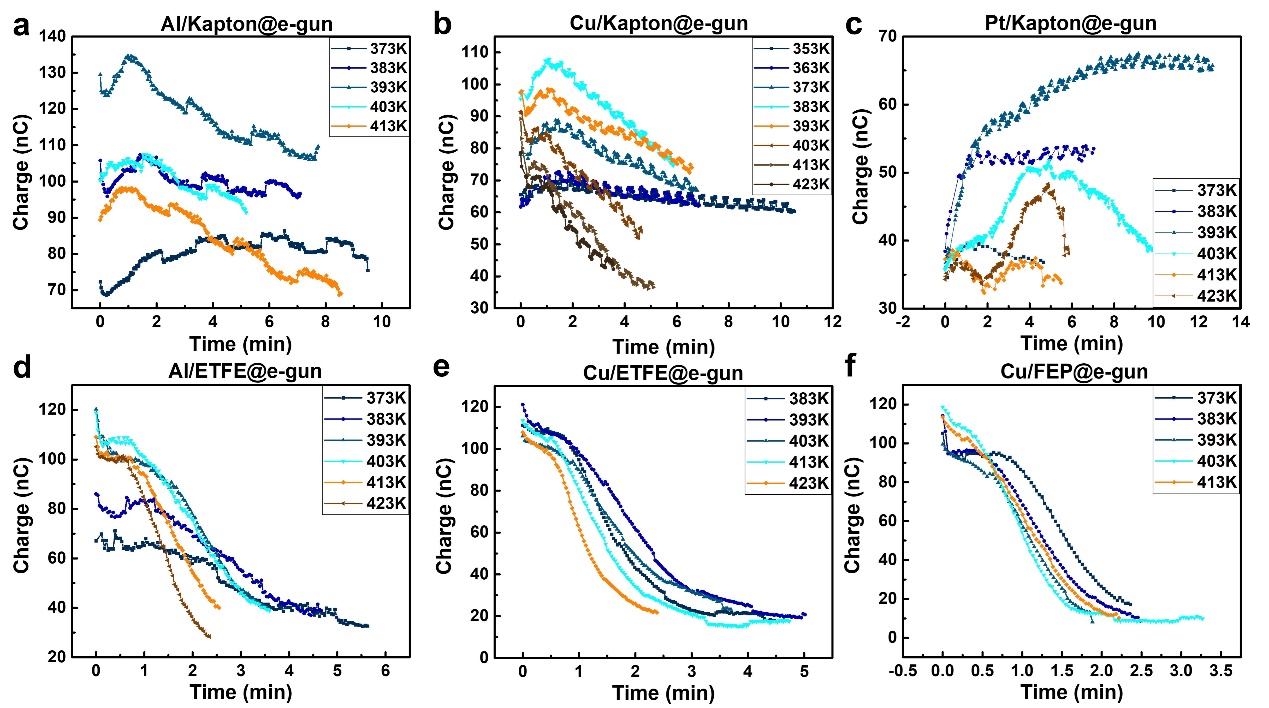


**Figure S6.** Charge evolution of different material pairs. **a**. Al/Kapton; **b**. Cu/Kapton; **c**. Pt/Kapton; **d**. Al/ETFE; **e**. Cu/ETFE; **f**. Cu/FEP. All the initial charge were induced by e-gun.


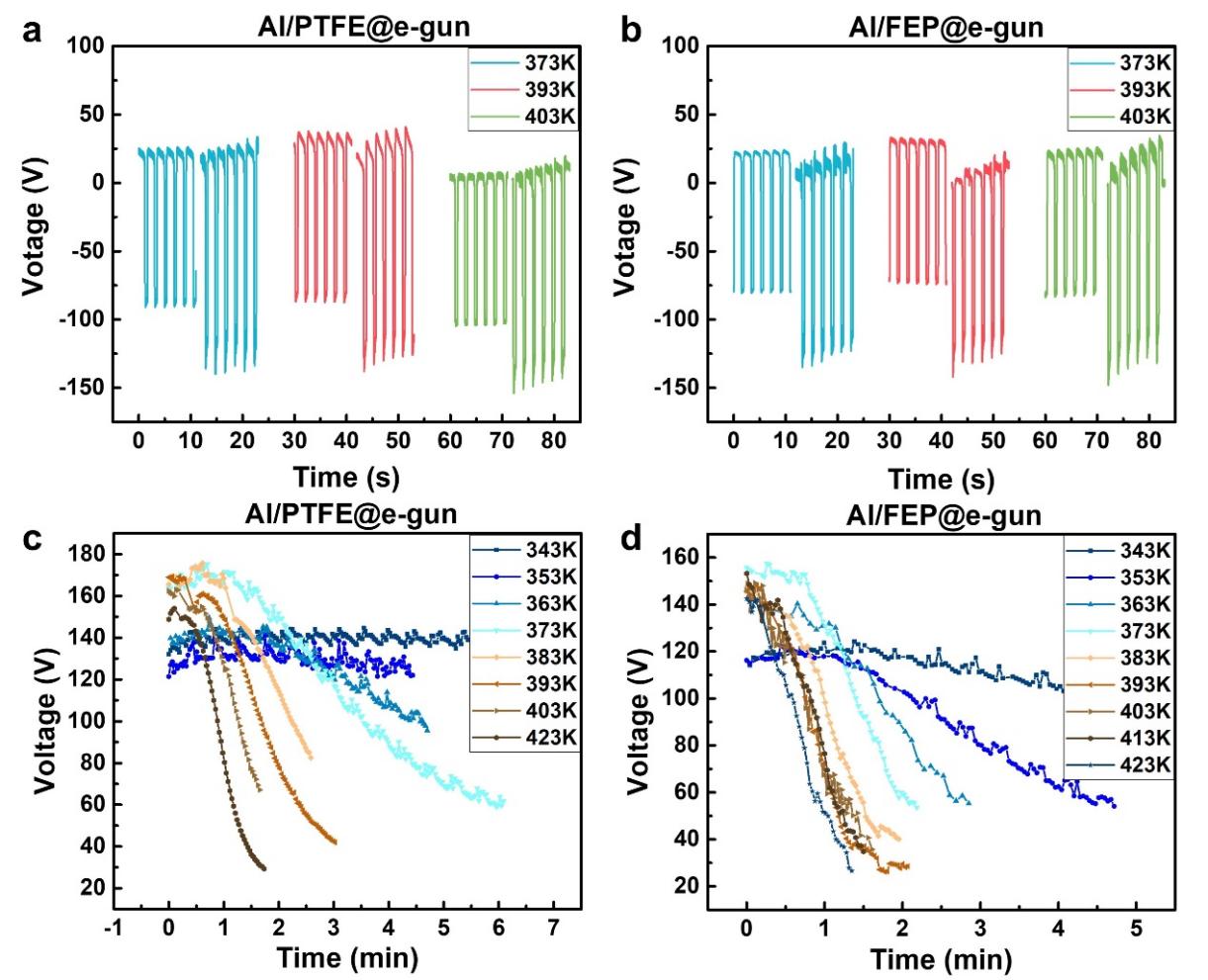


**Figure S7.** Voltage variation by the heat-excitation effect. Voltage comparison of **a.** Al/PTFE and **b.** Al/FEP at different temperature. Voltage evolution against time of **c.** Al/PTFE and **d.** Al/FEP. The initial charge was induced by e-gun.


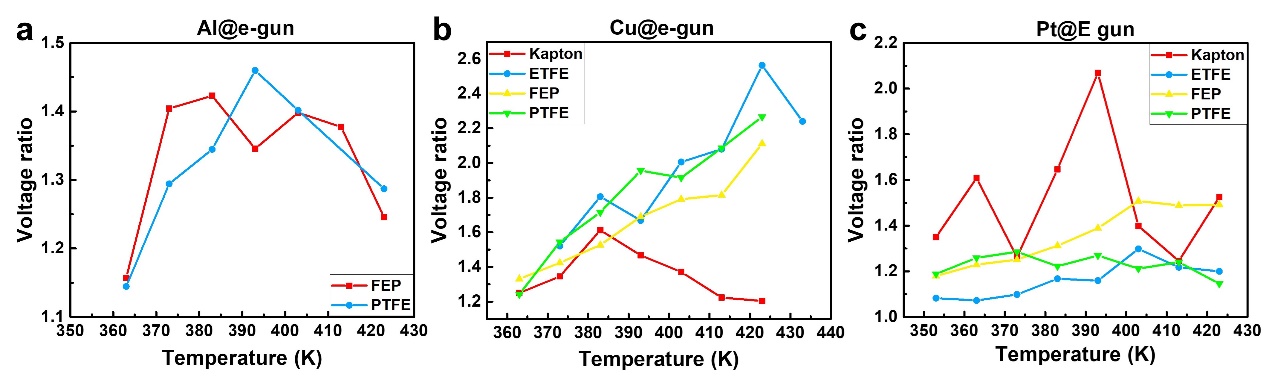


**Figure S8.** Voltage ratio of metals contacting with different polymers by the heat-excitation effect. **a**. Al; **b**. Cu; **c**. Pt.


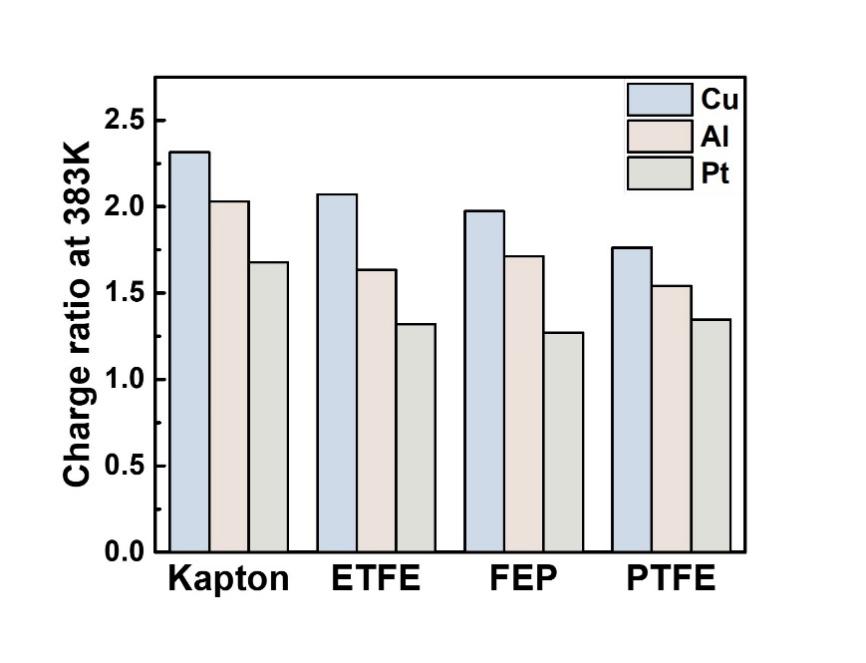


**Figure S9.** Excitation charge ratio at 383 K.


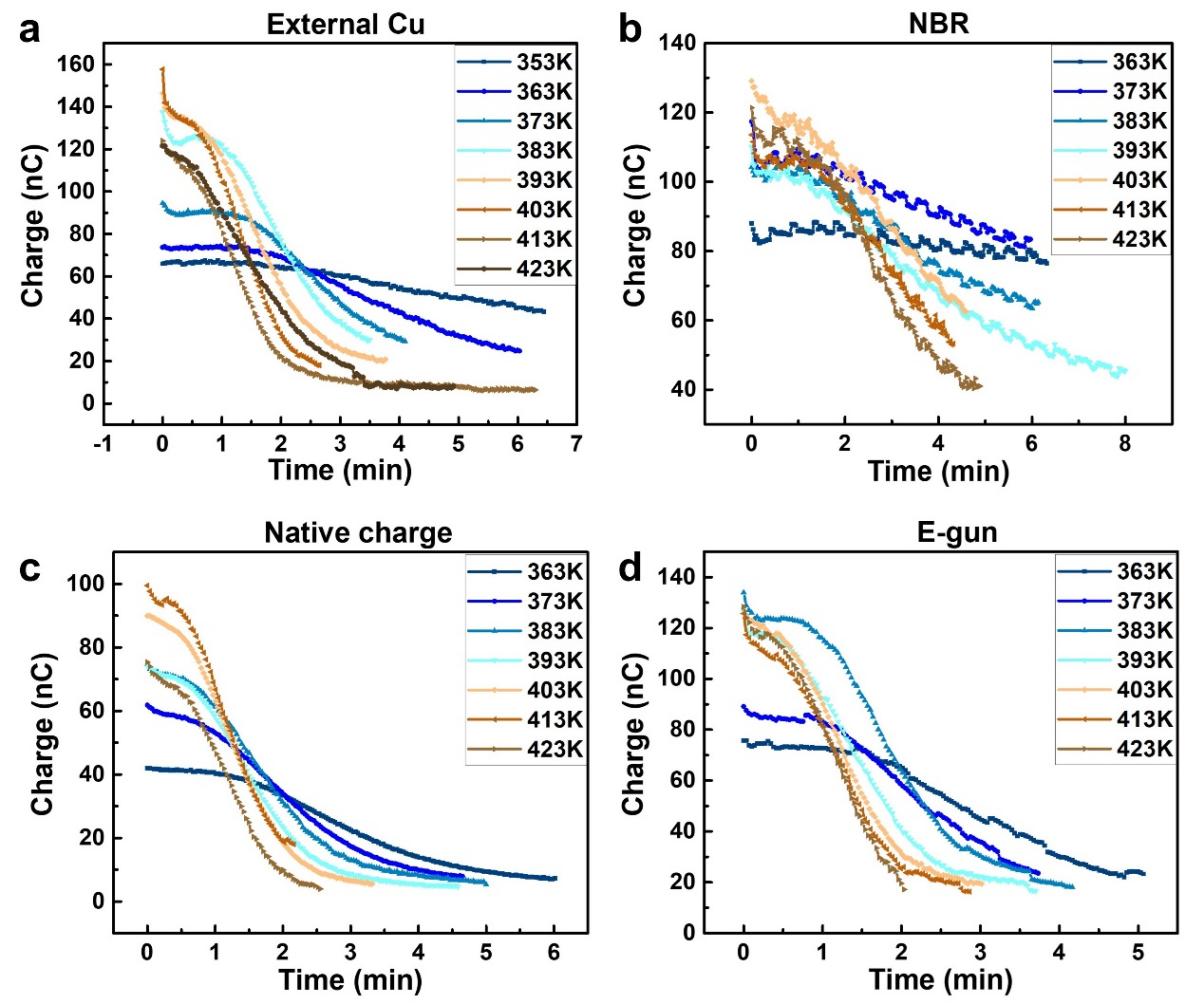


**Figure S10.** Charge evolution of Cu/FEP with different initial charge inducing methods. **a**. External Cu; **b**. NBR; **c**. Native charge; **d**. E-gun.


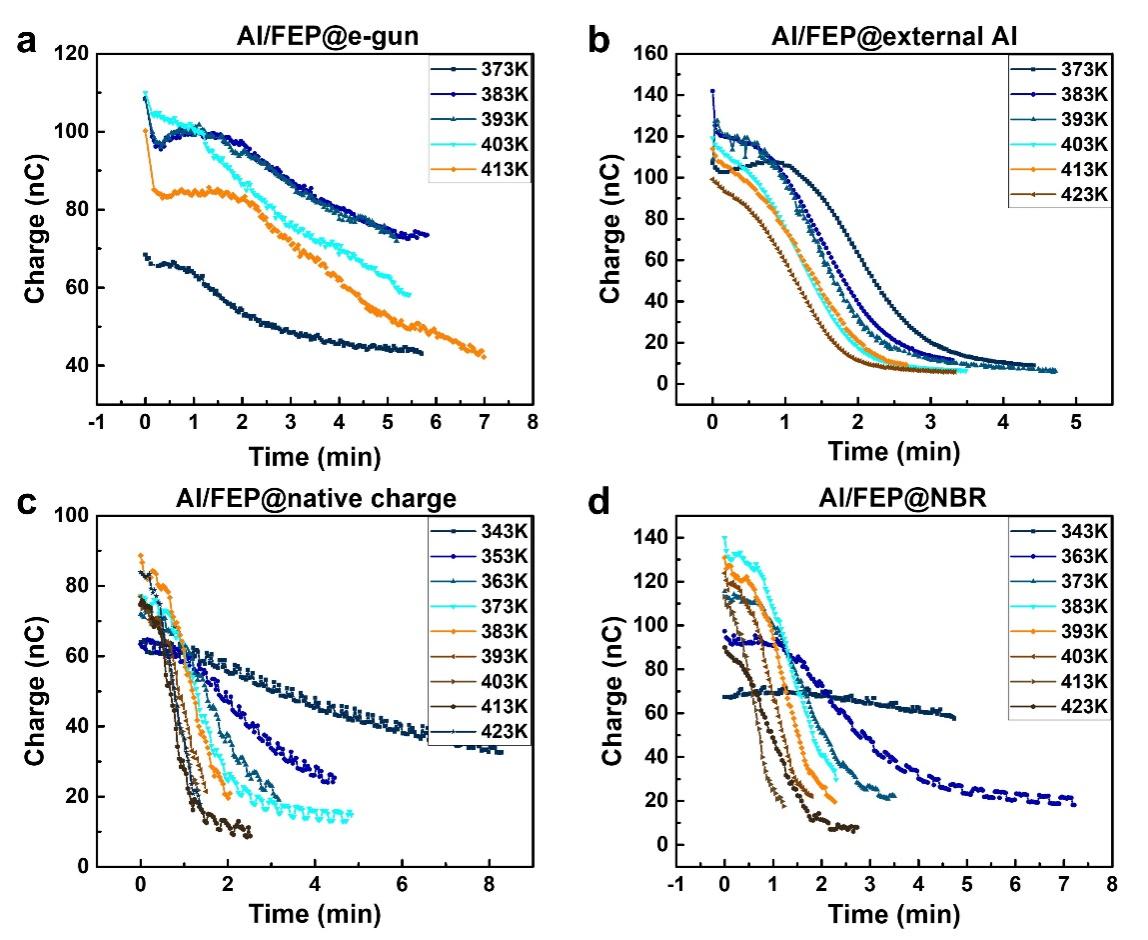


**Figure S11.** Charge evolution of Al/FEP with different initial charge inducing methods. **a**. E-gu; **b**. External Al; **c**. Native charge; **d**. NBR.


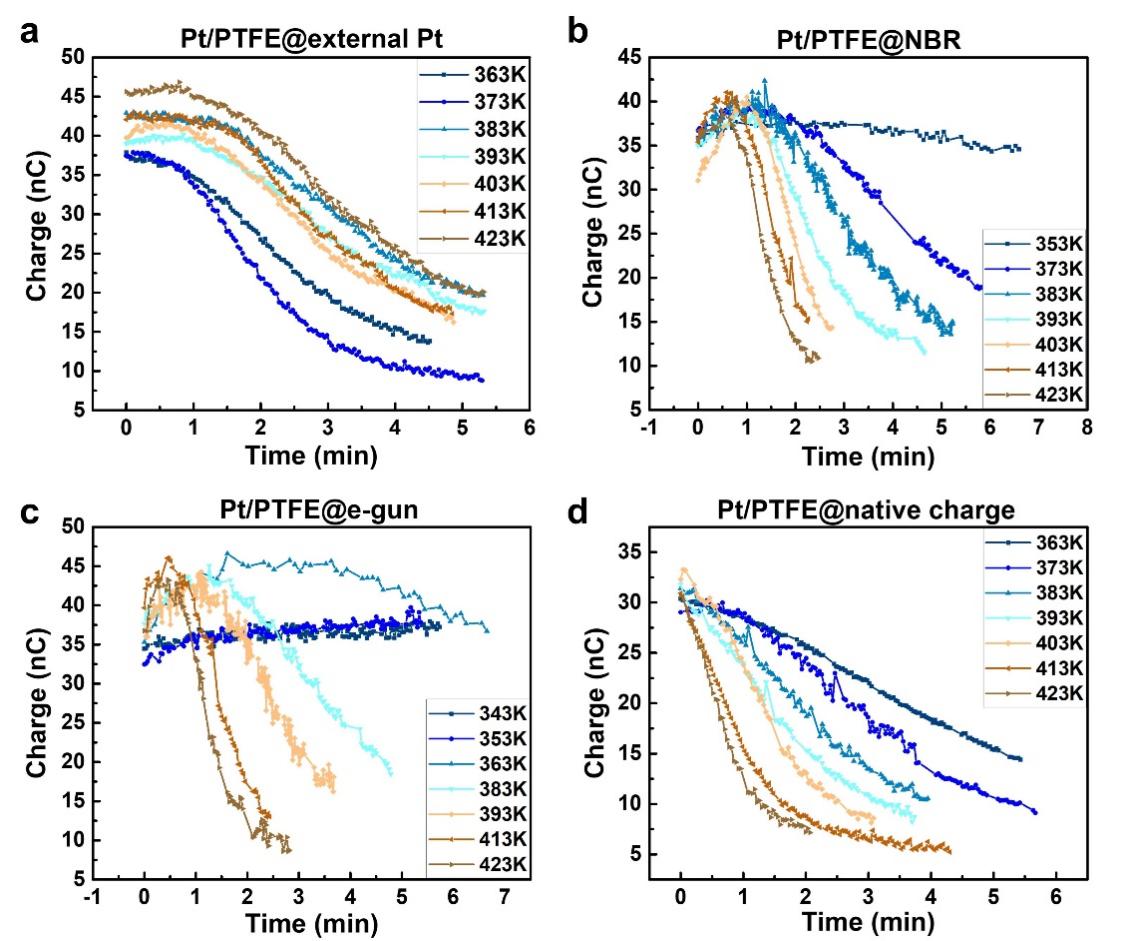


**Figure S12.** Charge evolution of Pt/PTFE with different initial charge inducing methods. **a**. External Pt; **b**. NBR; **c**. E-gun; **d**. Native charge.


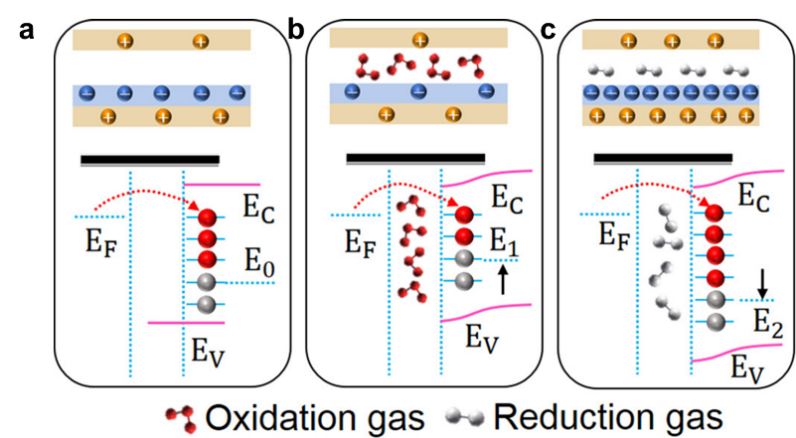


**Figure S13**. The surface state model. **a**. CE between the metal and the polymer. **b**. CE in metal/polymer pair with O_3_ at the interface. **c**. CE in metal/polymer pair with H_2_ at the interface. Reproduced with permission, Copyright 2020, American Chemical Society^[43]^.


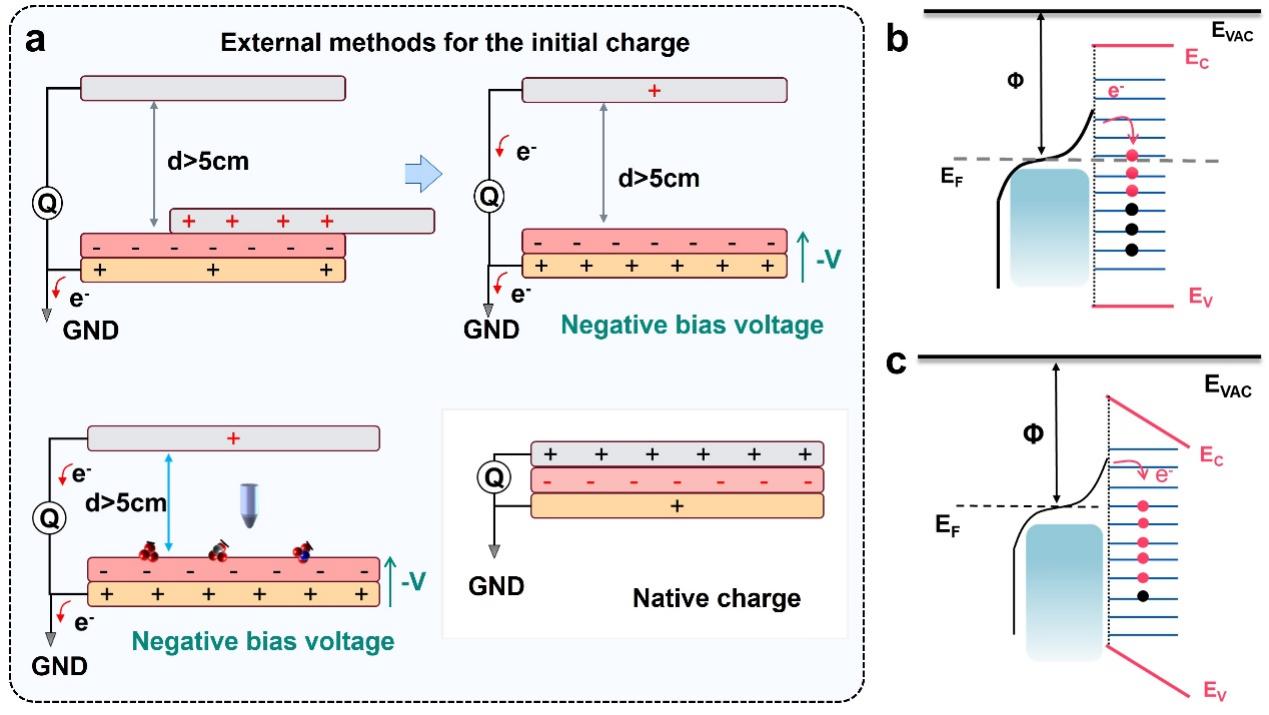


**Figure S14.** Charge evolution of Pt/PTFE with different initial charge inducing methods. **a**. charge transfer model of external methods. Energy band model without **b**. electric filed bias and **c**. with negative electric field bias.


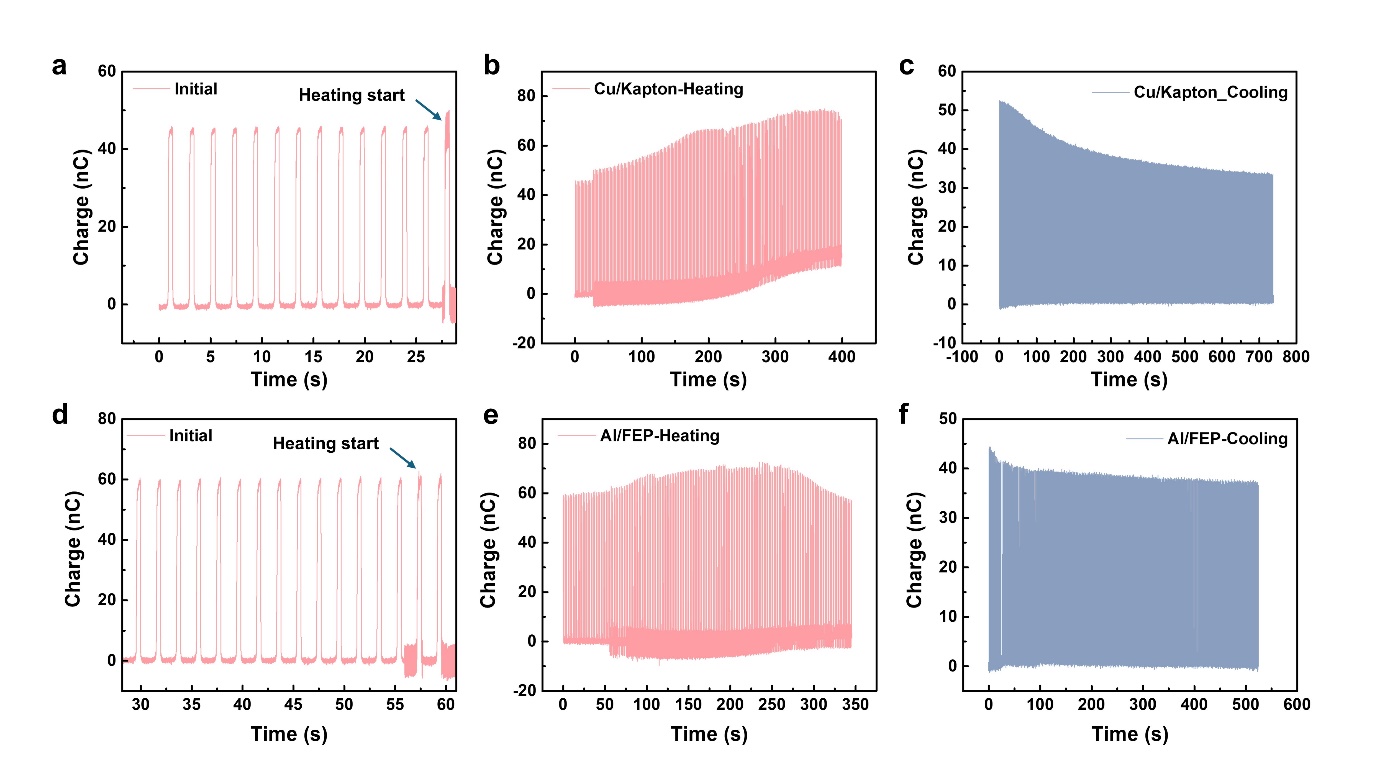


**Figure S15.** Heating and cooling behaviors of **a-c.** Cu/Kapton and **d-f**. Al/FEP.


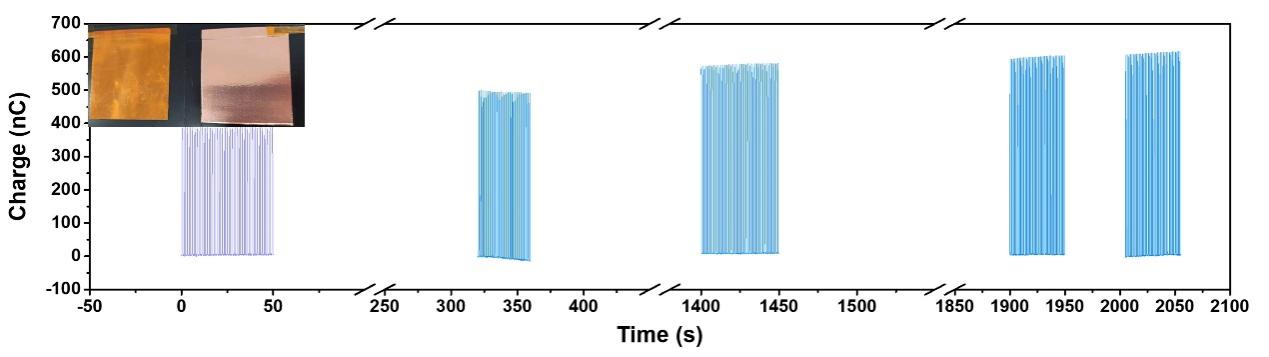


**Figure S16.** Durability test of 7cm-by-7cm Al/Kapton device with silicone substrate at 373K.


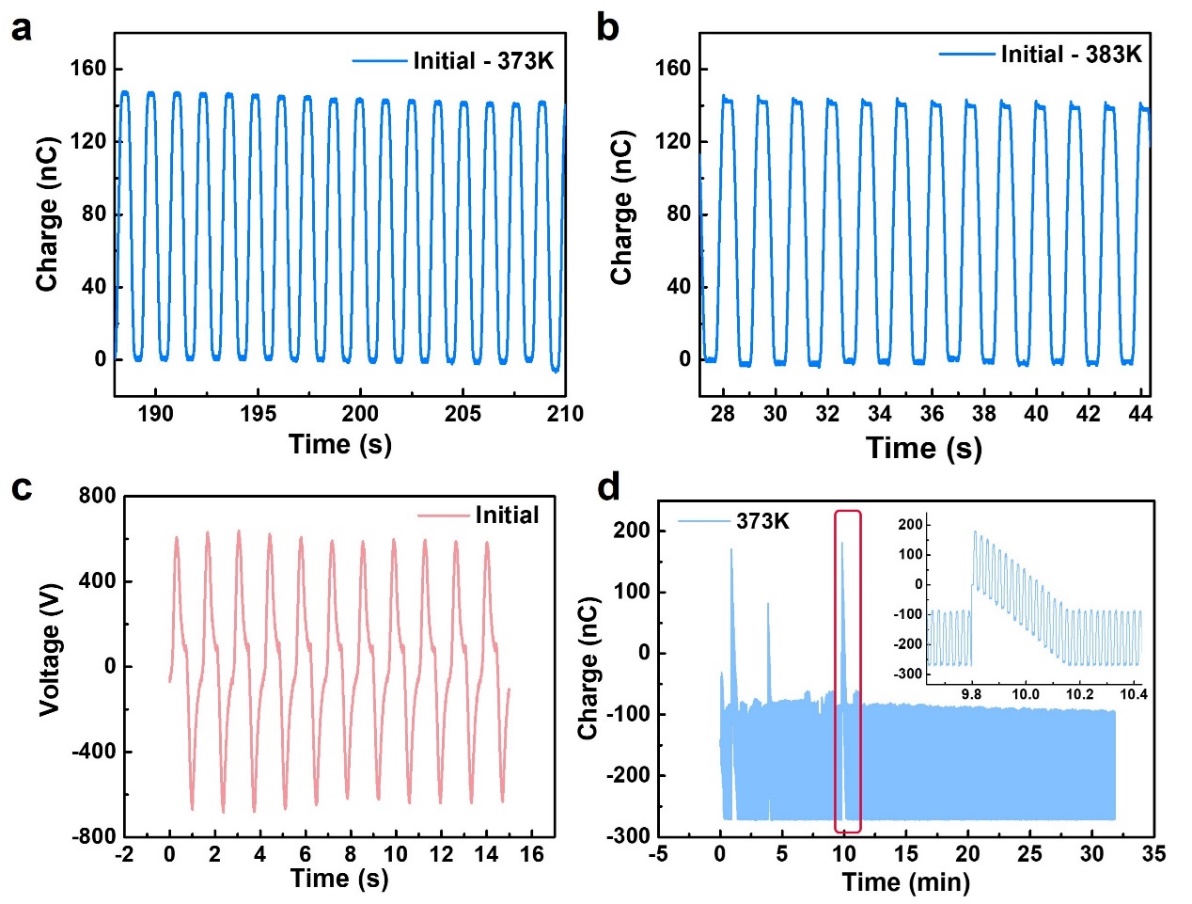


**Figure S17.** Electrical performance of SFT mode TENG with discharger. Initial charge of **a.** 373K and **b.** 383K. **c.** Initial voltage of 383K. **d.** Charge durability of 373K. The fluctuation in the inset of **d.** came from the charge drift by *Keithley 6514*.


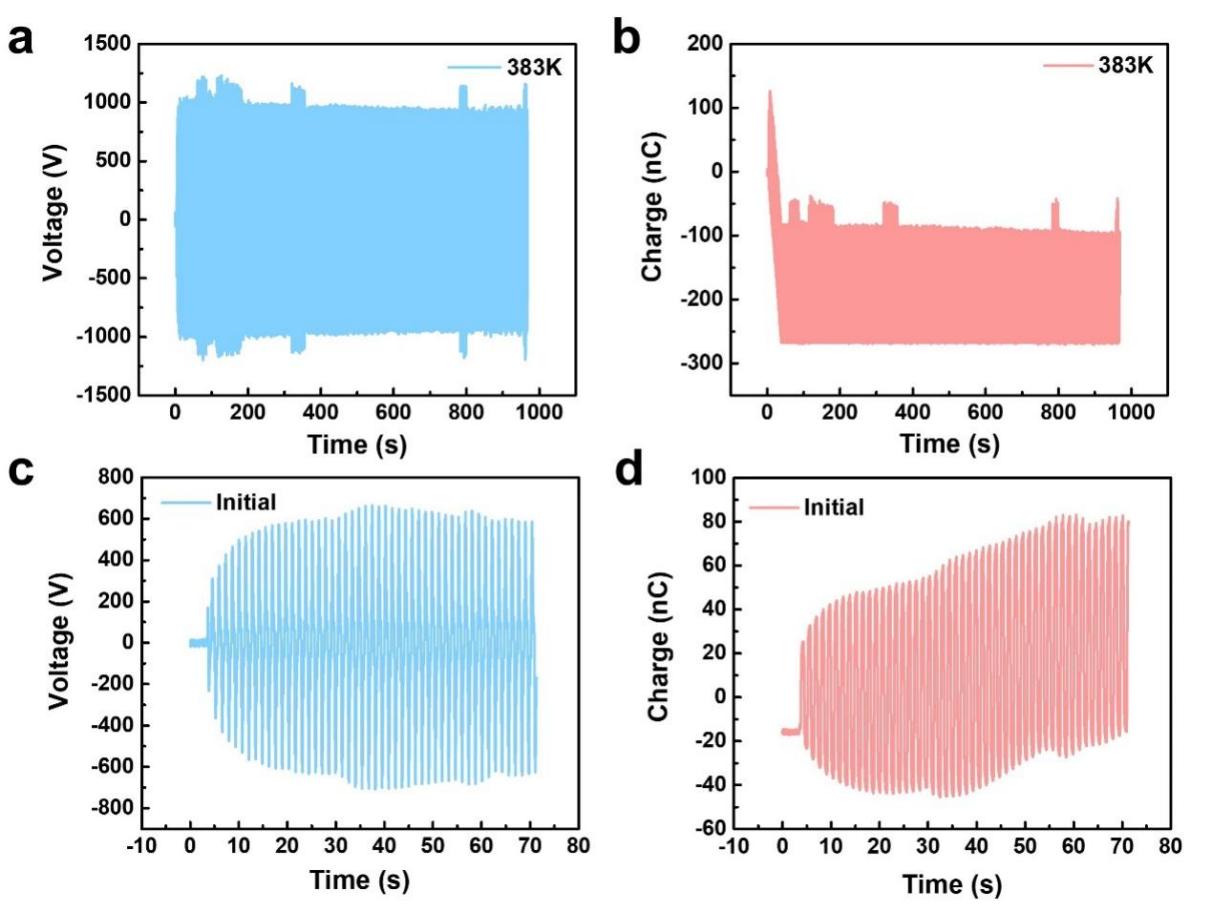


**Figure S18.** Electrical performance of CMEO measurement. **a.** voltage and **b.** charge durability test at 383K. **c.** Initial voltage and **d.** charge output.


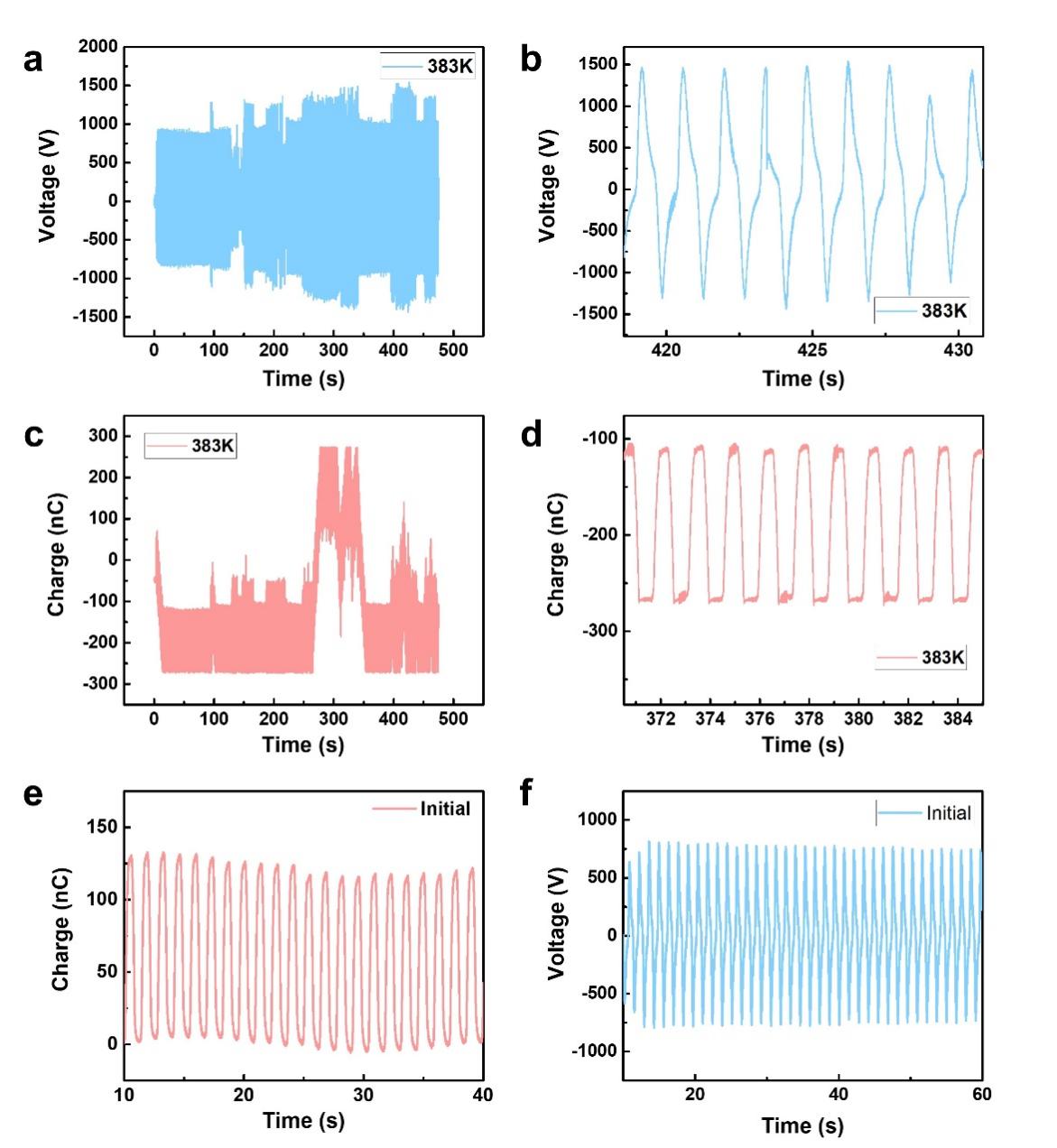


**Figure S19.** Electrical signals of SFT mode TENG with discharger. **a.** Voltage and **b.** charge durability test at 383K. **c.** Initial charge and **d.** voltage output.


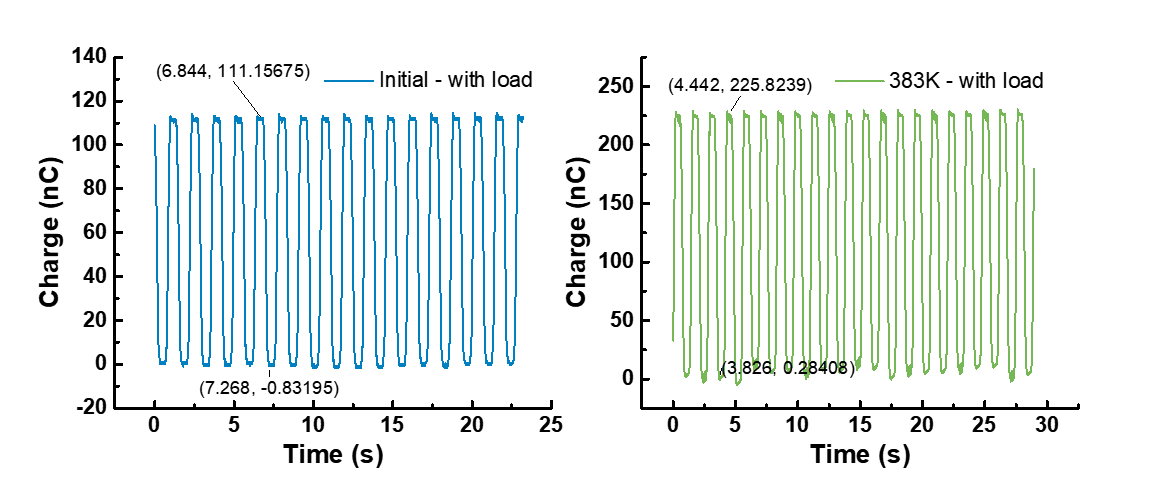


**Figure S20.** Charge output for capacitor charging.

**Note S1.** Charge transfer processes of metal/polymer pair in the static measurement.


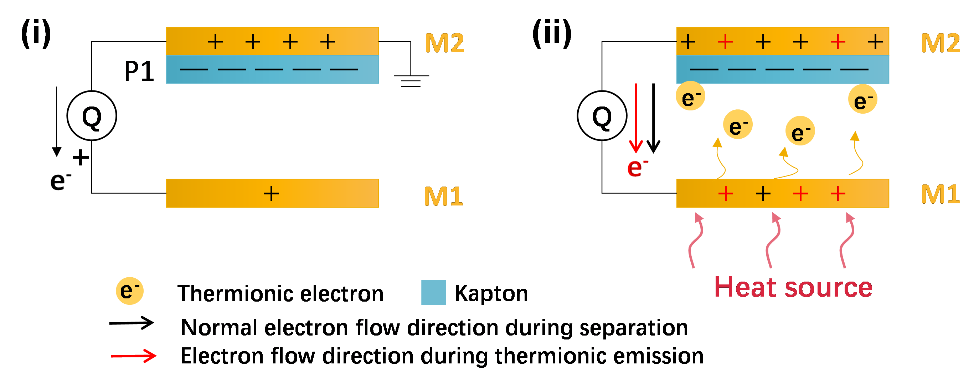


**Figure S21.** Static measurement method with heater at the positive side. **i.** Normal charge transfer process during separation. **ii.** Charge transfer processes during thermionic emission.

Assume the initial surface charge is *Q*_SC_, and the thermionic emission electrons are Δ*Q*_SC_. During the separation process, electrons transfer from M2 to M1 (**i**). When the heater was located at the positive side, the electrons were emitted from the M1 surface with charge amount of Δ*Q*_SC_. Thus, the charge evolution was listed as follows:

M1: surface charge was enhanced from *Q*_SC_ to *Q*_SC_+Δ*Q*_SC_ because of the thermionic emission.

M2: surface charge was enhanced from *Q*_SC_ to *Q*_SC_ to *Q*_SC_+*α*Δ*Q*_SC_. Here, *α* is the ratio that the thermionic electrons partially gathered at the polymer side.

Therefore, electrons should be transferred from M2 to M1 with amount of *α*Δ*Q*_SC_ to realize the new electrostatic equilibrium. The static flow should be the same the initial flow. However, the results from Al(heated)/Kapton reflected an opposite charge flow, indicating that the thermionic emission did not happen at the M1 surface but the polymer side.

If the thermionic emission was the dominant reason for the charge enhancement by the heat-excitation, the final net charge output should be *Q*_SC_+*α*Δ*Q*_SC_, that means *αQ*_SC_ = *Q*_SC_(*T*)- *Q*_SC_(*T*_0_)

For the Al (heated) /Kapton pair at *T*=413K, the charge output was enhanced from 55nC to 95nC, indicating that the *α*Δ*Q*_SC_ should be at least 40nC. However, the measured charge variation was only around 2 nC, demonstrating that the charge enhancement should not come from the thermionic emission.
